# Tissue-specific directionality of cellulose synthase complex movement inferred from cellulose microfibril polarity in secondary cell walls of Arabidopsis

**DOI:** 10.1038/s41598-023-48545-z

**Published:** 2023-12-12

**Authors:** Juseok Choi, Mohamadamin Makarem, Chonghan Lee, Jongcheol Lee, Sarah Kiemle, Daniel J. Cosgrove, Seong H. Kim

**Affiliations:** 1 Department of Chemical Engineering, Materials Research Institute, Pennsylvania State University, University Park, PA 16802 USA; 2 Department of Computer Science and Engineering, Pennsylvania State University, University Park, PA 16802 USA; 3 Materials Characterization Laboratory, Pennsylvania State University, University Park, PA 16802 USA; 4 Department of Biology, Pennsylvania State University, University Park, PA 16802 USA

**Keywords:** Plant sciences, Physical chemistry

## Abstract

In plant cells, cellulose synthase complexes (CSCs) are nanoscale machines that synthesize and extrude crystalline cellulose microfibrils (CMFs) into the apoplast where CMFs are assembled with other matrix polymers into specific structures. We report the tissue-specific directionality of CSC movements of the xylem and interfascicular fiber walls of *Arabidopsis* stems, inferred from the polarity of CMFs determined using vibrational sum frequency generation spectroscopy. CMFs in xylems are deposited in an unidirectionally biased pattern with their alignment axes tilted about 25° off the stem axis, while interfascicular fibers are bidirectional and highly aligned along the longitudinal axis of the stem. These structures are compatible with the design of fiber-reinforced composites for tubular conduit and support pillar, respectively, suggesting that during cell development, CSC movement is regulated to produce wall structures optimized for cell-specific functions.

## Introduction

**Figure 1:**
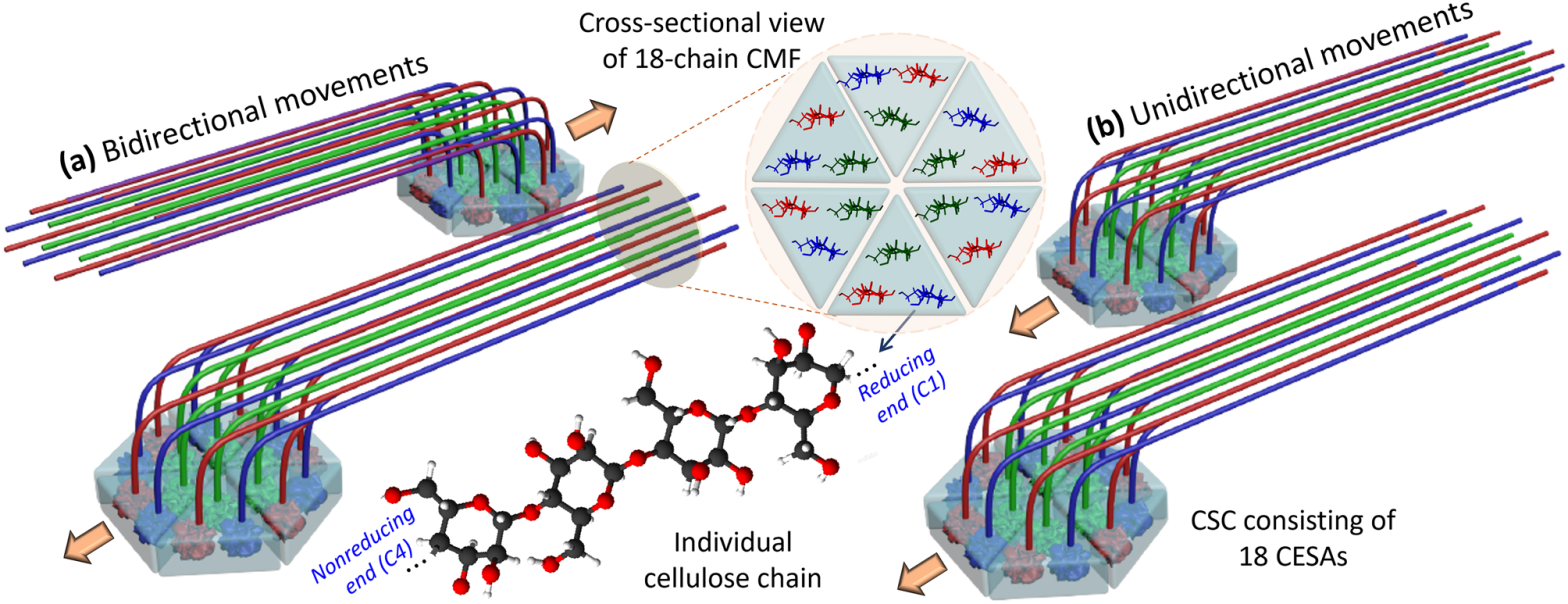
Schematic illustration of (**a**) bidirectional and (**b**) unidirectional movements of CSCs extruding CMFs along the movement track (not shown).

Cellulose in plant cell walls plays critical roles for plant structure and growth^1,2^. Cellulose is considered the most abundant renewable biopolymer on earth and its technical applications extend far beyond conventional papers, textiles, and food additives^3,4^. In plants, cellulose is produced by cellulose synthase (CESA) proteins in the plasma membrane which are assembled into ~ 30 nm diameter clusters with a six-fold symmetry, which are called cellulose synthase complex (CSC)^5–8^. During cellulose synthesis, monomeric glucose units are supplied to the CSC from the cytoplasm side and the synthesized polymer chains are extruded into the apoplast^9^. Due to the proximity facilitating hydrogen bonding and van der Waals interactions among them^10^, the chains extruded from individual CESAs in CSCs aggregate into a cellulose microfibril (CMF; see Fig. 1). Depending on the degree of interchain packing within CMF and inter-fibril packing, CMFs can have a crystalline order. These CSCs are then combined with other matrix polymers to form cell walls with specific physical structures that vary depending on the requisite function^11,12^. Thus, the mesoscale packing pattern of CMFs is likely linked to the tissue-specific cell wall function during the plant growth.

Since the already-extruded CMF portion is fixed into the wall matrix, the subsequent elongation of CMF through polymerization causes CSC to move forward within the plasma membrane^2,13^. The CSC movements during the deposition of CMFs have been tracked in vivo via optical imaging of fluorescence-tagged CESAs^13^. Since this method works only for the optically-accessible exterior surface of the plant, most in situ imaging of live cells has been carried out with epidermal walls of hypocotyls^14,15^. Many fluorescence imaging studies found that the orientation distribution of the CSC movements is highly correlated with that of the microtubules underneath the plasma membrane^14–17^, although other steering mechanisms were also reported^18^. However, the key parameters and mechanism to determine CSC directionality are still unclear. For example, several studies reported that CSC movements were bidirectional along the microtubule axis (Fig. 1a)^12,13^. But, in a recent study where secondary cell walls were transdifferentiated in the epidermis of *Arabidopsis* hypocotyl, it was found that CSCs move in clusters and their moving directions are preferentially biased (Fig. 1b)^12^.

The observation of different trends in primary cell walls and induced secondary cell walls raise an important question that has not been studied before. Does the CSC movement directionality vary with cell type? Is it related to the biological or physical function of each cell wall? If so, it may mean that the CMF deposition process is structurally engineered to produce the wall structure that is optimized to carry specific functions of cell in each tissue region^19,20^.

Here, we report the CSC movements in secondary cell walls inside the plant deduced from the postmortem analysis of the already-deposited CMFs in individual cell walls. Because the polymerization reaction in CESAs is the regiospecific insertion of the reducing end (C1 position) of the monomeric glucose unit to the non-reducing end (C4 position of the last monomeric unit) of the cellulose chain^21,22^, the CMFs extruded from CSCs have a polarity. If CSCs move bidirectionally with equal probabilities along the movement axis, the C1 → C4 directionality of the cellulose chains among adjacent CMFs will be antiparallel on average (Fig. 1a). The unidirectional movement of CSCs will result in the parallel C1→ C4 directionality among adjacent CMFs (Fig. 1b). Once CFMs are deposited and imbedded in the amorphous matrix, their polarity cannot be reverted during the post-deposition stretching or expansion of the cell wall. Thus, the polarity of CMF in the cell wall can be related to the directionality of CSC movement during the cell growth. Note that the mechanism and factors controlling the CSC movement directionality is beyond the scope of this study.

To differentiate the CMF polarity, we have used vibrational sum frequency generation (SFG) microscopy. SFG is a second-order optical process where an infrared photon is mixed with an up-conversion photon (conveniently, called visible) and a new photon is emitted at the frequency which is the sum of the two input photons^23,24^. Being a non-linear optical spectroscopy, SFG requires non-centrosymmetry over a space defined by the coherence length of this non-linear optical process^23^. In the plant cell wall, crystalline cellulose is the only non-centrosymmetric component fulfilling this requirement; all other matrix polymers (e.g. hemicellulose, lignin, etc.) form the amorphous matrix in which CMFs are distributed. Thus, SFG can selectively detect crystalline CMFs in plant cell walls without interferences from other amorphous components^25–27^. The coherence length can be as small as tens of nanometers in a reflection-SFG experiment and as large as tens of micrometers in a transmission experiment^28–30^. The relative intensities of the CH and OH stretch modes of cellulose (centered at 2944 cm^–1^ and 3320 cm^–1^, respectively) are associated with the orientation as well as directionality of CMFs^30–34^. Using the dipole moment orientations of these modes with respect to the CMF axis, the SFG intensities at 2944 cm^–1^ and 3320 cm^–1^ can be theoretically calculated as a function of CMF orientation and directionality^35^. Combining hyperspectral SFG imaging^34^ with the SFG theory^35^, we were able to determine the orientation and directionality of CMFs in xylem and interfascicular fiber (IFF) walls inside an *Arabidopsis* inflorescence stem.

## Results

### Hyperspectral SFG imaging of 8-week-old Arabidopsis inflorescence stem

**Figure 2:**
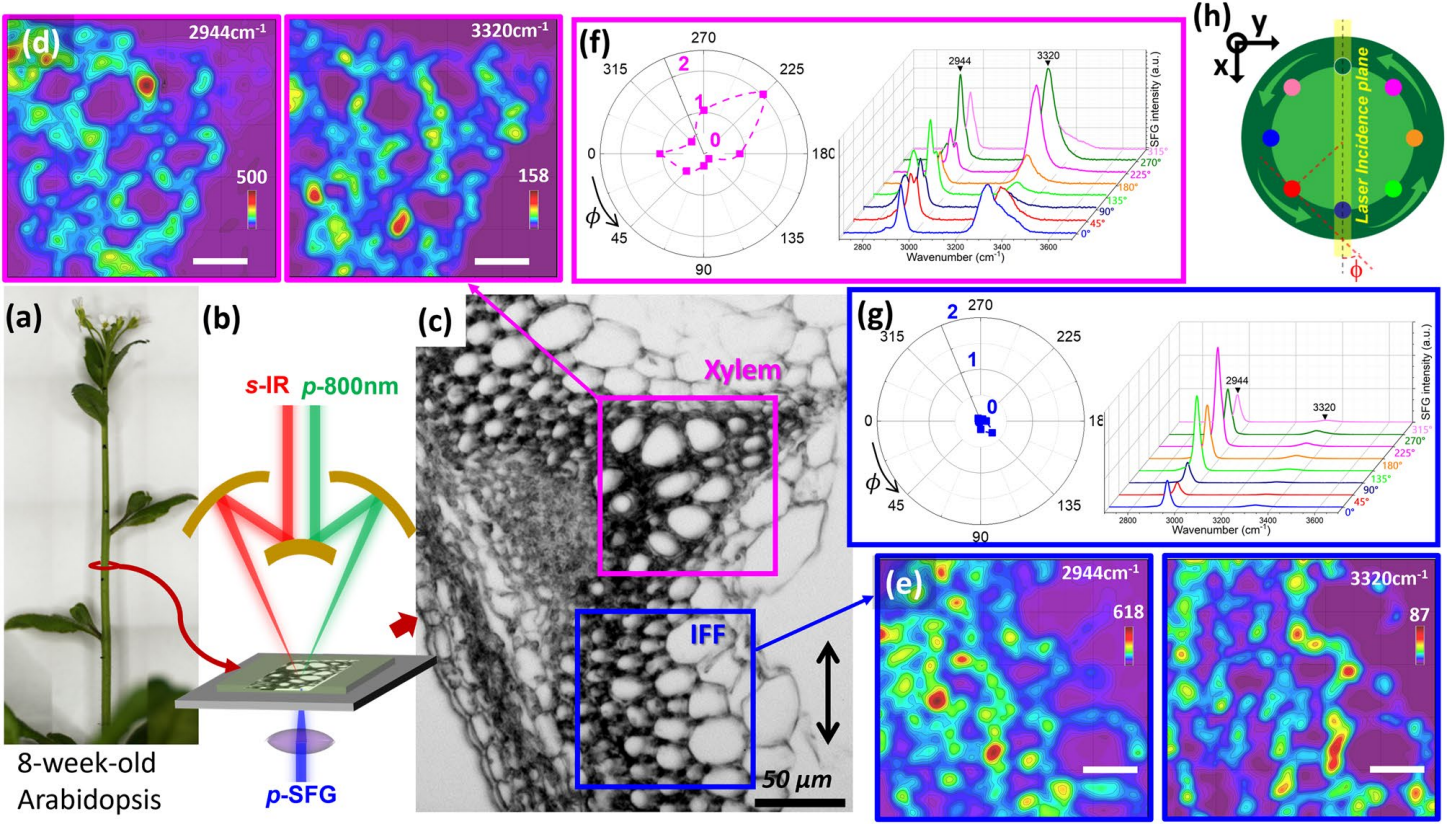
A picture of 8-week-old *Arabidopsis* inflorescence stem and microscopic SFG analysis result. (**a**) A ~ 10 μm thick sectioned sample was placed on a slide glass and covered with a ~ 130 μm thick cover glass with D_2_O in the space between the two glasses. (**b**) The emitted SFG signal, 800 nm up-conversion beam, and incident IR beam had *p-*, *p-*, and *s-*polarizations, respectively, with respect to the laser incidence plane defined by the two incidence beams (marked with a black arrow above the scale bar in the optical microscope image (**c**))*.* The hyperspectral intensity maps of 2944 cm^–1^ and 3320 cm^–1^ are shown for (**d**) xylem and (**e**) IFF regions marked with pink and blue boxes, respectively, in the optical image. The spatial resolution of SFG microscopy was ~ 2.5 μm along the laser incidence plane, ~ 4 μm normal to the laser incidence plane, and ~ 15 μm into the depth direction^34^. (**f**,**g**) The SFG spectra at 8 locations around one single cell wall in the xylem and IFF regions, extracted from the hyperspectral image, are shown in the insets. The polar plot in the inset shows how the intensity ratio of the 3320 cm^–1^ OH peak versus the 2944 cm^–1^ CH peak varies as a function of azimuth angle (ϕ). As shown in (**h**), the azimuth angle is defined by the angle between the tangential line of each data point and the laser incidence plane. Two other sections of biological replicates are shown in Fig. S3.

The cell walls inside the inflorescence stem of an 8-week-old *Arabidopsis* were exposed through transverse sectioning and analyzed with SFG spectroscopy (Fig. 2a–c). Note that SFG cannot determine the directionality of individual CMFs, as it relies on the interference of adjacent CMFs within the coherence length and its spatial resolution is only on the order of micrometers. Instead, SFG differentiates the overall polarity and average orientation of CMFs within the probe volume^30^. Also, since the lateral resolution is larger than the thickness of S1, S2, and S3 layers within the secondary cell wall, SFG data represents the average of those three layers which is predominantly influenced by the S2 layer because it is much thicker than the S1 and S3 layers^36^. The 2944 cm^–1^ and 3320 cm^–1^ intensity maps extracted from hyperspectral images of cellulose in the xylem and IFF regions are shown in Fig. 2d and e. These are two peaks characteristic to cellulose in the CH and OH stretch regions, respectively^27^. Also shown are SFG spectra extracted from eight locations around one cell wall in the xylem and IFF region of the hyperspectral image in Fig. 2f and g. In the xylem region, the 3320 cm^–1^ intensity varied drastically along the wall of a single cell, and its intensity was as big as the 2944 cm^–1^ peak at certain locations. The walls of two adjacent xylem cells were often found to have different SFG spectral features (see Fig. S1). In contrast, the 3320 cm^–1^ peak of the IFF wall was significantly smaller than the 2944 cm^–1^ peak, regardless of the location. The tilt angle of transition dipole of the 2944 cm^–1^ (CH) and 3320 cm^–1^ (OH) modes with respect to the c-axis of cellulose Iβ unit cell is ~ 62° and ~ 35°, respectively^27^. Because the SFG signals of vibrational modes with dipole angles above and below the magic angle (54.7°) vary differently with the azimuth orientation, their relative intensities are quite informative in the analysis of CMF arrangement in mesoscale.

### Theoretical prediction of azimuth and tilt angle dependence of cellulose SFG signal

Even though SFG microscopy does not have a nanoscale spatial resolution, the spectral features extracted from individual pixels can provide the nanoscale structural information of CMFs, especially the average orientation and directionality^30^. In order to quantitatively deconvolute the directionality and orientation of CMFs inside the cell wall exposed by transverse sectioning of the stem and placed vertically along the optical axis (Fig. 2), comparison of the experimental data with the intensities theoretically calculated for given geometric patterns is necessary^35^. For this comparison, the relative intensity ratio of the two peaks with different dipole orientations (i.e. 3320 cm^–1^/2944 cm^–1^), instead of their individual intensities, was used; in this way, intensity variations due to light scattering from rough sample textures could be cancelled out. The CMF polarity can be defined with the degree of directional excess (*DE*):

$$DE= \frac{\mathrm{difference \,\,between \,\,two \,\,opposite \,\,directions \,\,along \,\,the \,\,alignment \,\,axis}}{\mathrm{sum \,\,of \,\,all\,\, CMFs \,\,within\,\, the \,\,probe \,\,volume}} \times 100\%.$$

**Figure 3:**
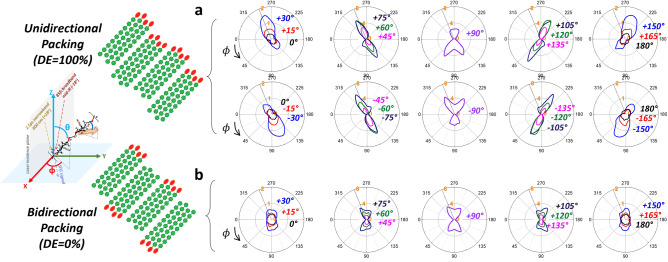
Polar plots of the calculated 3320 cm^–1^/2944 cm^–1^ *pps-*SFG intensity ratio (OH/CH) as a function of azimuth angle (ϕ) at selected tilt angle (θ; shown as numbers in each plot) for (**a**) unidirectional (*DE* = 100%) and (**b**) bidirectional (*DE* = 0%) packing of CMFs. The angles are defined with respect to the lab coordinate as schematically illustrated; the cross-sectioned sample is placed in the XY plane, and the laser incidence plane is in the XZ plane. The scale of each plot is marked with orange color numbers. In the numerical calculation, the CMF diameter is assumed to be 4 nm and the inter-CMF distance is 5 nm, which corresponds to 40 vol.% of cellulose (typical cellulose content in secondary cell wall)^30,38,39^ and is close to the experimentally measured values for woody tissues^40,41^. Here, both ϕ and θ angles are assumed to have a Gaussian probability function with a standard deviation (σ) of 10° and no specific rotational preference of crystallographic facets about the CMF axis. The calculation results for different σ_ϕ_ and σ_θ_ are shown in Fig. S2. Details of calculation can be found in Ref.^35^.

Figure 3 displays polar plots of the calculated 3320 cm^–1^/2944 cm^–1^ intensity ratio for two cases – unidirectional packing (DE = 100%) vs. bidirectional packing (DE = 0%) – as a function of the azimuth angle (ϕ) between the uniaxial alignment axis of CMF and the laser incidence plane for selected sets of tilt angle (θ) of CMF with respect to the surface normal direction (which is the objective lens axis of the microscope). Since the stem is transversely sectioned and placed on the horizontal (XY) plane, the tilt angle (θ) in Fig. 3 is equivalent to the microfibril angle (MFA) used in wood science^37^. The data shown in Fig. 3 are the calculated results assuming the Gaussian probability function for both ϕ and θ with a standard deviation (σ) of 10°.

When CMFs are deposited with *DE* = 100% (Fig. 3a), the azimuth angle dependence of the 3320 cm^–1^/2944 cm^–1^ intensity ratio is asymmetric in four quadrants of the ϕ-polar plot. The exception is at θ  = 90°; when CMFs are parallel to the XY plane, the polar plot show a mirror plane along the Y-axis (θ = 90° and 270°) with different amplitudes depending on whether the C1→C4 direction of the cellulose chain is pointing toward the positive or negative direction of the Y-axis. A similar trend was observed in the SFG analysis of a unidirectionally deposited bacterial cellulose although detailed patterns were slightly difference because bacterial cellulose has mostly Iα allomorph^42^. In other tilt angles (ϕ ≠ 90°), which quadrant of the polar plot has a larger value varies depending on whether the C1→C4 direction of CMF is tilted clockwise (θ > 0°) or counterclockwise (θ < 0°).

When CMFs are deposited with *DE* = 0% (Fig. 3b), the azimuth polar plot of the 3320 cm^–1^/2944 cm^–1^ intensity ratio exhibits a mirror plane along the X-axis (ϕ = 0° and 180°), although some minor details may differ. When CMFs are parallel to the XY plane (i.e. θ = 90°), the polar plot shows a two-fold rotational symmetry about the Z axis. In the case of partial deviation from the bidirectional polarity (i.e. *DE* = 50%), the azimuth polar plot of the 3320 cm^–1^/2944 cm^–1^ intensity ratio displays intermediate patterns between these two extreme cases (See Fig. S2).

These calculation results lay out theoretical foundation that can be used to the orientation (θ) and polarity (DE) of CMFs within the microscale probe area. Relying on the comparison with theoretical models is the main difference of SFG microscopy from conventional techniques such as scanning electron microscopy (SEM), transmission electron microscopy (TEM), and atomic force microscopy (AFM) which rely on nanoscale spatial resolution to resolve individual CMFs. And, none of these conventional imaging methods can provide the polarity information of CMF, although they can provide the orientational distributions of individual CMFs^19,43^.

### Azimuth angle dependence of cellulose SFG signal from hyperspectral imaging

Since the cell shape is relatively circular, the tangential line of each pixel around a single cell can be used to define the azimuth angle (ϕ) of CMFs at that location with respect to the laser incidence plane. The 2944 cm^–1^ and 3320 cm^–1^ intensities at various locations of the wall around a single cell can be read from the hyperspectral images and their ratios are plotted as a function of azimuth angle in Fig. 2. More data extracted from the same section sample as well as two other section samples are also shown in Fig. S3.

In the xylem region, the ϕ-polar plot of the 3320 cm^–1^/2944 cm^–1^ intensity ratios show the asymmetric pattern with values large in one quadrant of the ϕ-polar plot. The comparison with the theoretical calculation results shown in Fig. 3 suggests that the DE of CMF in the xylem wall is not nearly zero, i.e. having a parallel polarity. The fact that the line profile across two cell walls contacting each other often showed different 3320 cm^–1^/2944 cm^–1^ ratios (Fig. S1**)** indicates that the CMF orientation and polarity even in the adjacent cells are different.

In the IFF region, the 3320 cm^–1^/2944 cm^–1^ ratio is very small (close to 0.1 in average) and does not show any discernable ϕ-dependence. The comparison with the theoretical calculation result (Fig. 3) suggested that the CMF tilt angle (θ) with respect to the stem axis must be close to zero. Although the theoretical simulation predicts a subtle difference in the ϕ-dependence between DE = 0 vs. 100% cases at near zero tilt angle (Fig. 3), in practice it is difficult to distinguish these two cases due to fluctuation in experimental data. Alternatively, this can be determined by analyzing the ϕ-dependence of the sample in which the CMFs are oriented perpendicular to the laser incidence plane (i.e. θ = 90°). Fig. S4 compares the ϕ-polar plot of the 2944 cm^–1^ and 3320 cm^–1^ SFG signal from the longitudinally sectioned stem with the theoretical predictions for DE = 0, 50, and 100% at θ = 90°. This comparison suggests that the DE of the CMF in the IFF region should be close to 0%, i.e. the antiparallel polarity overall.

### Tilt angle and directionality of CMFs in xylem and IFF

The theoretical calculation data predicting the 3320 cm^–1^/2944 cm^–1^ ratio as a function of ϕ, θ, and DE can be used as a training data set for a multi-layer perceptron (MLP) neural network algorithm to find the correlation model; once the model is constructed and tested, the ϕ-polar plot data extracted from the SFG hyperspectral images of the transverse cross-section samples can be processed to determine the θ and DE of CMFs in each cell wall. In the transmission SFG analysis, the OH/CH intensity ratio is attenuated due to absorption loss of IR input beam, and the scattering loss of SFG signal by the medium is larger in the OH stretch region^44^. Assuming such attenuations do not vary with θ and DE of CMF in the sample, the experimental data can still be compared with the theoretically predicted patterns.

**Figure 4:**
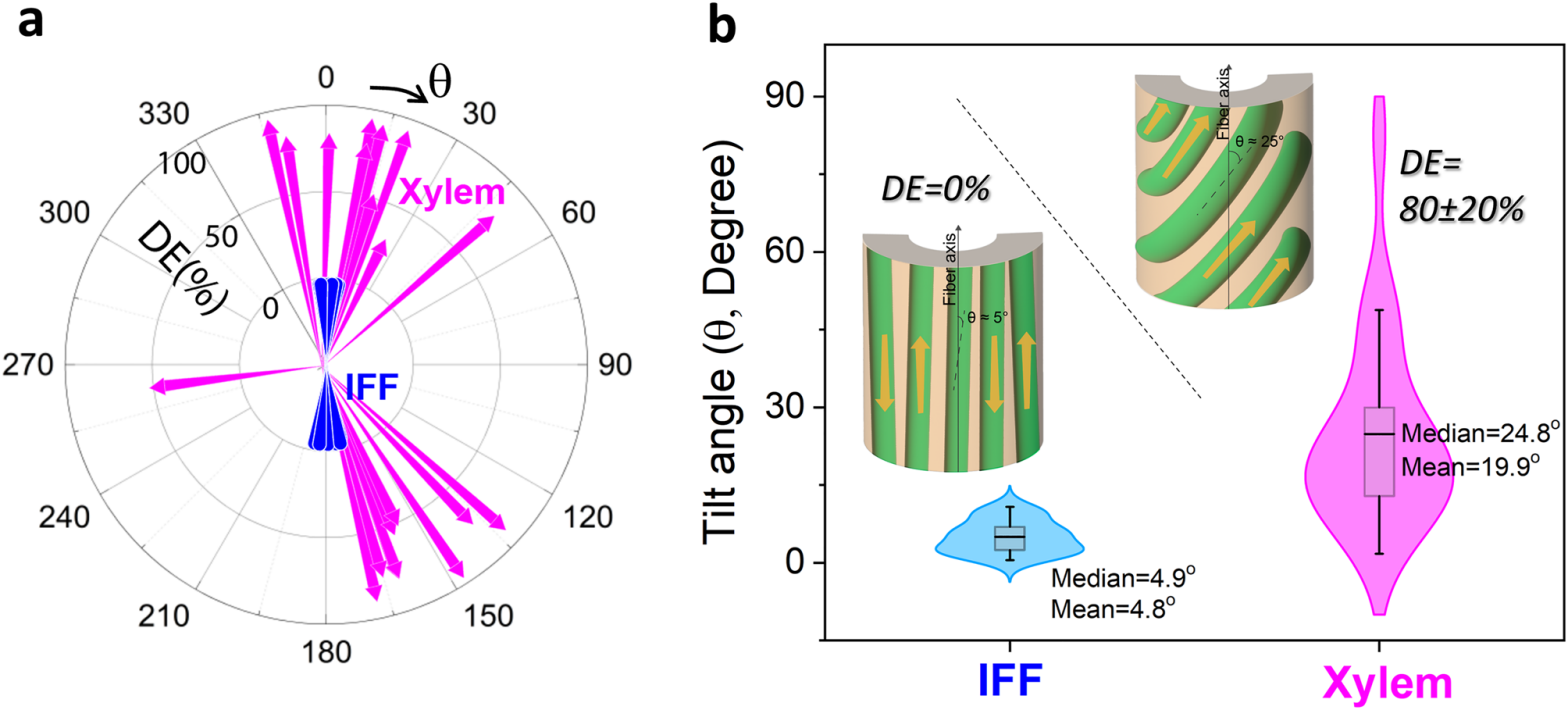
(**a**) Polar plot of tilt angle (θ) and polarity (DE) of CMF, determined via the MLP analysis of azimuth angle (ϕ) dependence of the 3320 cm^–1^/2944 cm^–1^ intensity ratio, for 20 IFF cells (blue cones) and 20 xylem cells (pink arrows) selected in the SFG hyperspectral images of three transverse cross-sections of 8-week-old *Arabidopsis* stem. The fit results of individual cell walls are shown in Fig. S5. In the polar plot, the inner circle is DE = 0% and the middle circle is DE = 100%, and the length of bar represents the DE value determined from the MLP analysis. (**b**) Distribution of CMF tilt angle (θ) in the IFF and xylem cell walls calculated from the data in (**a**). The inset drawings are an artistic rendition of the θ and DE distributions determined from the SFG analysis.

Figure 4a shows the tilt angle (θ) and polarity (DE) of CMF determined from the ϕ-dependence of the SFG signals for 20 IFF cell walls and 20 xylem cell walls. In the case of IFF, only the simulation data shown in Fig. 3b were used as the training set since the CMF polarity was already determined to be DE = 0% (Fig. S4), leaving only one degree of freedom (θ) to be determined. This analysis showed that in all 20 IFF, CMFs are highly aligned along the stem axis. The mean MFA is calculated to be ~ 5° (Fig. 4b), which is in good agreement with a previous study conducted with scanning x-ray microdiffraction^40^.

In the case of xylem, all ϕ dependencies simulated for DE = 0%, 50%, and 100% (Fig. 3 and Fig. S2) were used as the training set, and both θ and DE were determined from the MLP analysis. The results showed that the MFA distribution of CMFs is relatively broad with a median value of 25° (Fig. 4b), and DE is on average 80% (standard deviation = 20% from N = 20). This implies that CSCs moved mostly in a highly unidirectional fashion when the xylem cell walls were produced. This result is congruent with the previous live cell imaging study in which fluorescence-tagged CESA movements in transdifferentiated xylem cell walls were monitored^14,45^. The previous SFG study of the transdifferentiated protoxylem walls of Arabidopsis also suggested the unidirectionally-biased polarity of CMFs^30^. Note that even two cell walls adjacent to each other have different tilt angles. In many cases, it was observed that one is winding up and the other is winding down within the same MFA range.

### Comparison with engineering principle of fiber-reinforced composite materials

We have compared the average MFA and DE of CMFs in individual IFF and xylem walls with the structural engineering and mechanics principles of fiber-reinforced polymer composite materials to see if the CSC movement during CMF synthesis and deposition is related to the tissue-specific function of the cell wall. In the stem, the IFF walls serve a mechanical function supporting the body weight of the plant^46^. In a fiber-reinforced polymer composite sheet, it is well known that the in-plane load bearing capacity is higher along the fiber direction^47^. The fact that CMFs in IFF walls are highly aligned along the vertical stem axis (Fig. 4b) confirmed that the IFF wall structure is optimally designed for the maximum capacity of compressive stress due to gravity.

The main function of xylem is the water transport from root to leaf via negative pressure produced by evapotranspiration; thus, the cell wall must have good resistance against vessel collapse^48^. If the CMF synthesis in the secondary cell wall is suppressed, then the xylem cells are collapsed due to the negative pressure gradient generated by evapotranspiration^48,49^. The engineering design for high resistance to out-of-plane collapse or rupture of a conduit is to have a spiral fiber reinforcement (Fig. 4b), as can be seen in a coil-reinforced hose^50^. Such a coiled structure in cell walls must be achieved by controlling the orientation of the CSC guiding track (such as microtubules or other steering mechanisms)^14–16,18,51^.

Another contributing mechanism that can facilitate the coiling process could be the coupling with the intrinsic structural property of CMF^52^. Each CMF can be twisted due to the chirality of the cellulose structure^53,54^. If CMFs in a macrofibril are aligned unidirectionally, then their strain energy could add up and eventually cause the macrofibril to coil^52^. If the macrofibril is composed with bidirectionally-packed CMFs, the strain energy of individual CMFs will cancel out internally and natural coiling will not occur. Such unidirectional packing of CMF requires the unidirectional movement of CSCs during the cellulose synthesis^42^, and it is not achievable through post-synthesis process of bidirectionally-deposited CMFs.

## Discussion

The MFA and polarity of CMFs in the xylem and IFF walls inside *Arabidopsis* stems were analyzed with vibrational SFG spectroscopy, from which the CSC movement during the CMF synthesis could be deduced. In the xylem wall, CMFs were found to be deposited in an unidirectionally biased pattern with their fibril axes tilted about 25° off the stem axis, which aligns with the engineering principle for the high resistance to out-of-plane collapse of a conduit under negative pressure gradient. In the IFF wall, CMFs are bidirectional and highly aligned along the longitudinal axis of the stem, which is ideal for the maximum compressive load bearing capacity along the gravity axis. These findings may suggest that plants produce the cell wall in a specific structure optimized for individual tissue function by regulating the CSC movement during the cell wall synthesis.

## Methods

### 8-week-old *Arabidopsis thaliana*

The ecotype Col-0 of *Arabidopsis thaliana* was used for all experiments. After 4 days of cold treatment at 4 °C, plants were grown on 1 × Murashige and Skoog (MS) medium^55^ containing 1% sucrose for one week, and transferred onto a soil mixture of sphagnum peat moss, vermiculite, perlite, and mycorrhizae (Pro-mix BX) at 22 °C/16 °C in a 12-h-light/12-h-dark photoperiod with 120–150 μE m^−2^ s^−1^ light exposure. Stems were harvested after 8 weeks of growth depending on the experiment. The stems were flash-frozen in Shandon™ Cryomatrix™ (Thermo Scientific), then cryo-sectioned into 20-μm-thick transverse sections using a Leica CM1950 cryostat, placed in water, and washed 3 times with 1 mL water. Then, the aqueous medium was fully replaced with D_2_O to avoid the IR attenuation in the OH stretch region by H_2_O. The cryo-sectioned sample was then sandwiched with a cover glass and a slide glass with D_2_O in between and then analyzed with SFG in the fully hydrated state^33^.

### SFG microscopy

A custom-designed SFG microscopy system used for this study has been fully described elsewhere^34,44^. In brief, an 800 nm up-conversion laser pulse and a tunable, broadband IR pulse were overlapped temporally and spatially on the sample. The 85 fs 800 nm laser pulses were generated with a Ti–Sapphire amplifier (Libra, Coherent) at a 2 kHz repetition rate. The IR pulses with a full width at half maximum of 100–150 cm^–1^ were generated with an optical parametric generator/optical parametric amplifier (OPG/OPA) system (OPerA Solo, Coherent) pumped with the 800 nm pulses. The 800 nm narrowband pulse for SFG spectroscopy was produced by stretching the 85 fs pulse (with a 12 nm band width) to 2.4 ps (resulting in a 0.76 nm band width) using two etalons. These pulses were focused onto the sample using a 36× reflective objective lens (NA = 0.52, Newport). The incident 800 nm and IR beams were *p*- and *s*-polarized with respect to the incidence plane and the emitted SFG signal was detected in the transmission mode at the *p*-polarization (Fig. 2). For hyperspectral imaging, the *Arabidopsis* cross-section sample was step-scanned with a 2 $\mu$ m step size at two IR broadband center at 2940 cm^–1^ for the CH stretch region and 3300 cm^-1^ for the OH stretch region. The collected broadband spectra at each pixel were processed with home-built software coded with *Mathematica* for data visualization.

### Multi-layer perceptron

The multi-layer perceptron (MLP) neural network algorithm^56^ was utilized to estimate the tilt angle (θ) and the degree of directional excess (DE) in a regression model. The network was composed of 4 fully connected layers with 4866 trainable parameters. The first layer took the ϕ-polar plot represented as a 1D vector as input, while the final layer predicted the value of θ and DE. To prevent overfitting due to limited training data, dropout regularization was applied after the activation on each layer. Additional training data was generated with 20% random errors to compensate for experimental measurements caused by unpredictable conditions. The model was trained for 5 epochs with a batch size of 16, utilizing the mean absolute error (MAE) loss function with the Adam optimizer to update the network parameters during backpropagation. The network training was performed on a hardware system with i9-9980XE CPU and Nvidia Titan RTX GPU.

### Supplementary Information

Supplementary Information 1.

Supplementary Information 2.

Supplementary Figures.

## Data Availability

All raw data used in the main text and supplementary materials are available in the Supporting information. The details for the simulation of SFG intensity can be found in Ref.^35^.
