# Supplementary material for: Tissue-specific directionality of cellulose synthase complex movement inferred from cellulose microfibril polarity in secondary cell walls of Arabidopsis
Source: Sci Rep. 2023 Dec 12;13:22007. doi: 10.1038/s41598-023-48545-z (PMC10716418; doi:10.1038/s41598-023-48545-z)
Supplement: Supplementary file 1 — Supplementary Information 1. [file 41598_2023_48545_MOESM1_ESM.pdf]

```
!pip install xlsxwriter
```

```
import torch
from torch import nn
from torch.utils.data import DataLoader
from sklearn.preprocessing import StandardScaler
import pandas as pd
import numpy as np
import math
import argparse
import xlsxwriter
```

```
torch.set_default_dtype(torch.float64)
```

```
class DEDataset(torch.utils.data.Dataset):
    def __init__(self, X, y, scale_data=False):
        if not torch.is_tensor(X) and not torch.is_tensor(y):
            #Apply scaling if necessary
            if scale_data:
                X = StandardScaler().fit_transform(X)
            self.X = torch.from_numpy(X)
            self.y = torch.from_numpy(y)

    def __len__(self):
        return len(self.X)

    def __getitem__(self, i):
        return self.X[i], self.y[i]


class MLP(nn.Module):
    def __init__(self):
        super().__init__()
        self.layers = nn.Sequential(
            nn.Linear(25, 64),
            nn.ReLU(),
            nn.Dropout(0.2),
            nn.Linear(64, 32),
            nn.ReLU(),
            nn.Dropout(0.2),
            nn.Linear(32, 32),
            nn.ReLU(),
            nn.Dropout(0.1),
            nn.Linear(32, 2) # 37 + 5
        )

    def forward(self, x):
        return self.layers(x)


class Simple1DCNN(nn.Module):
    def __init__(self):
        super(Simple1DCNN, self).__init__()
        self.layer1 = nn.Conv1d(in_channels=1, out_channels=20, kernel_size=5)
        self.act1 = nn.ReLU()
        self.layer2 = nn.Conv1d(in_channels=20, out_channels=10, kernel_size=1)
    def forward(self, x):
        x = self.layer1(x)
        x = self.act1(x)
        x = self.layer2(x)


class ConvNet(nn.Module):
    def __init__(self, input_size, output_size, kernel_size=5, stride=1, padding=2):
        super(ConvNet, self).__init__()
        self.conv1 = nn.Conv1d(input_size, 8, kernel_size=kernel_size, stride=stride, padding=padding)
        self.conv2 = nn.Conv1d(8, 16, kernel_size=kernel_size, stride=stride, padding=padding)
        self.fc1 = nn.Linear(16*25, 128)
        self.dropout = nn.Dropout(0.2)
        self.fc2 = nn.Linear(128, output_size)
        self.relu = nn.ReLU()

    def forward(self, x):
        x = x.unsqueeze(1)
```

```
        x = self.conv1(x)
        x = self.relu(x)
        x = self.conv2(x)
        x = self.relu(x)
        x = x.view(x.size(0), -1)
        x = self.fc1(x)
        x = self.relu(x)
        x = self.dropout(x)
        x = self.fc2(x)
        return x


class SequenceRegressor(nn.Module):
    def __init__(self, input_size, hidden_size, num_layers, output_size):
        super(SequenceRegressor, self).__init__()
        self.hidden_size = hidden_size
        self.num_layers = num_layers
        self.lstm = nn.LSTM(input_size, hidden_size, num_layers, batch_first=True)
        self.fc = nn.Linear(hidden_size, output_size)

    def forward(self, x):
        x = x.unsqueeze(2)
        # Initialize hidden and cell states
        h0 = torch.zeros(self.num_layers, x.size(0), self.hidden_size).to(x.device)
        c0 = torch.zeros(self.num_layers, x.size(0), self.hidden_size).to(x.device)

        # Forward propagate the input through the LSTM layers
        out, _ = self.lstm(x, (h0, c0))

        # Pass the final hidden state through the fully connected layer
        out = self.fc(out[:, -1, :])

        return out


def excel_to_list(exp_number, DE_list, de_scale, xls_list, tilt_scale, val=False):
    X = []
    y = []

    for i in xls_list:
        xls = pd.ExcelFile('./train_' + str(exp_number) + '/' + i)

        for k, de in enumerate(DE_list):
            azimuth = []
            df = pd.read_excel(xls, 'DE'+str(k*de_scale)) if not val else pd.read_excel(xls, DE_list[k])

            tilt_angle = [i*tilt_scale for i in range(int(360/tilt_scale))] if not val else [i for i in range(int(360/tilt_scale))]
            for i in tilt_angle:
                azimuth.append(df[i].values)

            for i in range(len(tilt_angle)):
                tilt = (tilt_angle[i] * math.pi) / 180.0 if not val else (tilt_angle[i] * tilt_scale  * math.pi) / 180.0
                de_norm = float(de_scale * k) / 100.0
                tilt_angle[i] = [tilt, de_norm]

            X = X + azimuth
            y = y + tilt_angle

    X = np.array(X)
    y = np.array(y)
    y = y.astype(float)

    return X, y


exp_number=1
val_number=4
val_count=51
sample_name='Xylem10%'
train=True
test=False

mlp = MLP()


torch.manual_seed(42)

# Load dataset
train_DE_list = [0, 25, 50, 75, 100]
```

```
    train_DE_list = [0, 25, 50, 75, 100]
    train_xls = ['train_'+str(i) + '.xlsx' for i in range(101)]

    X, y = excel_to_list(exp_number, train_DE_list, 25, train_xls, 10)

    # Prepare dataset
    train_dataset = DEDataset(X, y)

    val_DE_list = ['A', 'B', 'C']
    #val_DE_list = [0, 25, 50, 75, 100]

    val_xls = ['val' + str(val_number) + '.xlsx']

    X, y = excel_to_list(exp_number, val_DE_list, 50, val_xls, 15, True)
    #X, y = excel_to_list(exp_number, val_DE_list, 25, val_xls, 10)
    valid_dataset = DEDataset(X, y)

    trainloader = DataLoader(train_dataset, batch_size=16, shuffle=True)
    validloader = DataLoader(valid_dataset, batch_size=24, shuffle=False)

    # Define the loss function and optimizer
    loss_function = nn.L1Loss()
    #loss_function = nn.MSELoss()

    optimizer = torch.optim.Adam(mlp.parameters(), lr=1e-3)

    best_loss = 100.0
    #mlp.to('cuda')
    mlp.train()
    # Run the training loop
    for epoch in range(0, 50): # 5 epochs at maximum

        # Set current loss value
        current_loss = 0.0

        # Iterate over the DataLoader for training data
        for i, data in enumerate(trainloader):

        # Get and prepare inputs
            inputs, targets = data
            inputs, targets = inputs.double(), targets.double()

            # Perform forward pass
            outputs = mlp(inputs)

            # Compute loss
            loss = loss_function(outputs, targets)

            # Perform backward pass
            loss.backward()

            # Perform optimization
            optimizer.step()

            # Zero the gradients
            optimizer.zero_grad()

            current_loss += loss.item()

        if epoch % 1 == 0:
            cur_loss = (current_loss / len(trainloader))
            #print('Train Loss after epoch %5d: %.12f' % (epoch, cur_loss))
            mlp.eval()
            val_current_loss = 0.0
            for i, data in enumerate(validloader):
                inputs, targets = data
                inputs, targets = inputs.double(), targets.double()
                outputs = mlp(inputs)

                # Compute loss
                val_loss = loss_function(outputs, targets)
                val_current_loss += val_loss.item()

            val_cur_loss = (val_current_loss / len(validloader))

            if best_loss > val_cur_loss:
                best_loss = val_cur_loss
                print('save model after epoch %5d: %.12f' % (epoch, best_loss))
```

```
            torch.save(mlp.state_dict(), './train_' + str(exp_number)+ '/best_checkpoint.bin')

        current_loss = 0.0

    # Process is complete.
    print('Training process has finished.')


    xls = pd.ExcelFile('./train_' + str(exp_number)+ '/val' + str(val_number)+ '.xlsx')
    df2 = pd.read_excel(xls, sample_name)

    sample_count = val_count
    y = [0 for i in range(sample_count)]
    X = []

    for i in range(sample_count):
        # X.append(df2[i].values)
        X.append(df2[i].values)

    X = np.array(X)
    y = np.array(y)
    y = y.astype(float)

    dataset = DEDataset(X, y)
    testloader = DataLoader(dataset, batch_size=val_count, shuffle=False)

    mlp.load_state_dict(torch.load('./train_' + str(exp_number)+ '/best_checkpoint.bin'))
    mlp.eval()

    workbook = xlsxwriter.Workbook('train_' + str(exp_number) + '_' + sample_name + '.xlsx')
    worksheet = workbook.add_worksheet(sample_name)
    row = 0
    col = 0

    for i, data in enumerate(testloader):
        # Get and prepare inputs
        inputs, targets = data
        inputs, targets = inputs.double(), targets.double()
        targets = targets.reshape((targets.shape[0], 1))

        # Perform forward pass
        outputs = mlp(inputs)
        #print(inputs)
        angles = (outputs[:, :1] * 180.0) / math.pi # angle
        des = (outputs[:, 1:2]) * 100.0             # DE

        for i, tup in enumerate(zip(angles, des)):
            print(tup[0].item(), tup[1].item())
            worksheet.write(row, col, tup[0].item())
            worksheet.write(row, col + 1, tup[1].item())
            row += 1

    workbook.close()
```
