# Supplementary material for: Tissue-specific directionality of cellulose synthase complex movement inferred from cellulose microfibril polarity in secondary cell walls of Arabidopsis
Source: Sci Rep. 2023 Dec 12;13:22007. doi: 10.1038/s41598-023-48545-z (PMC10716418; doi:10.1038/s41598-023-48545-z)
Supplement: Supplementary file 2 — Supplementary Information 2. [file 41598_2023_48545_MOESM2_ESM.pdf]

In[ ]:=
```
ClearAll["Global`*"]
```

Input: Laser conditions

c : Speed of light (m/s)
lambda: wavelength (m)
Iangle: Incidence angel of IR, Vis, and SFG
Rangle: refractive angel of IR, Vis, and SFG (calclulated by Snell's law)

nwater: Refractive index in the medium 1 (water or air)
ncellulose: Refractive index in the medium 2 (cellulose)

In[ ]:=
```
c = 299 792 458 ;
lambdaIR = 3500 * 10^-9;
lambdaVis = 800 * 10^-9;
lambdaSFG = (lambdaIR * lambdaVis) / (lambdaIR + lambdaVis)  // N;
nwaterIR = 1.371; (*water at 3500nm *)
nwaterVis = 1.3290; (*water at 800nm *)
nwaterSFG = 1.3310; (*water at 650nm *)
nCelluloseIR = 1.4579; (*IR*)
nCelluloseVis = 1.464;  (*Vis*)
nCelluloseSFG = 1.467;  (*SFG*)

IangleVis = 18; (*degrees*)
IangleIR = -18; (*degrees;  + for co-propagaion, 1 for couner-propagation*)

RangleVis = -IangleVis;
RangleIR = -IangleIR;

(*snells law:n_air sin(teta_air)=n_cellulose sin(teta_cellulose)*)
TangleIR = ArcSin[nwaterIR * Sin[IangleIR Degree] / nCelluloseIR ] * 180 / π ;
(*transmission IR angle*)
TangleVis = ArcSin[nwaterVis * Sin[IangleVis Degree] / nCelluloseVis ] * 180 / π ;
(*transmission VIS angle*)

(*phase matching along x-dirextion for relfection*)
RangleSFG =
  -ArcSin[(nwaterVis * (c / lambdaVis) * Sin[IangleVis Degree] + nwaterIR * (c / lambdaIR) *
         Sin[IangleIR Degree]) / (nwaterSFG * (c / lambdaSFG))] * 180 / π;
(*phase matching along x-direction condition for transmission*)
TangleSFG = ArcSin[
    (nCelluloseVis * (c / lambdaVis) * Sin[TangleVis Degree] + nCelluloseIR * (c / lambdaIR) *
        Sin[TangleIR Degree]) / (nCelluloseSFG * (c / lambdaSFG))] * 180 / π ;
IangleSFG = -RangleSFG;
```

```
Piecewise[{{medium = "Air", nwaterIR == 1}, {medium = "Water", nwaterIR > 1}}];
Print["Medium = ", medium]
Piecewise[
  {{mode = "Co-propagating", IangleIR > 0}, {mode = "Counter-propagating", IangleIR < 0}}];
Print["Progagating = ", mode]

Print["Incident Angle of SFG, Vis, and IR = ", {IangleSFG, IangleVis, IangleIR}]
Print["Reflection Angle of SFG, Vis, and IR = ", {RangleSFG, RangleVis, RangleIR}]
Print["Transmisson Angle of SFG, Vis, and IR = ", {TangleSFG, TangleVis, TangleIR}]


(*phase mismatch along z-direction upon reflection*)
kr1 = (2 π * nwaterIR / lambdaIR) * Cos[IangleIR Degree];
kr2 = (2 π * nwaterVis / lambdaVis) * Cos[IangleVis Degree];
kr3 = (2 π * nwaterVis / lambdaSFG) * Cos[IangleSFG Degree];
dkr = kr3 + (kr1 + kr2) (*Reflection*);
Lcr = π / dkr;
Print["Coherence Length (nm, Reflection) = ", Lcr * 10^9, " nm"]
(*phase mismatch along z-direction for transmission*)
kt1 = (2 π * nCelluloseIR / lambdaIR) * Cos[TangleIR Degree];
kt2 = (2 π * nCelluloseVis / lambdaVis) * Cos[TangleVis Degree];
kt3 = (2 π * nCelluloseSFG / lambdaSFG) * Cos[TangleSFG Degree];
dkt = kt3 - (kt1 + kt2) (*Transmission*);
Lct = π / dkt;
Print["Coherence Length (um, Transmission)= ", Lct * 10^6, " um"]
```

```
Medium = Water

Progagating = Counter-propagating

Incident Angle of SFG, Vis, and IR = {11.0653, 18, -18}

Reflection Angle of SFG, Vis, and IR = {-11.0653, -18, 18}

Transmisson Angle of SFG, Vis, and IR = {10.0283, 16.2913, -16.8937}

Coherence Length (nm, Reflection) = 126.406 nm

Coherence Length (um, Transmission)= 7.88841 um
```

Fresnel Coefficient (Reflection vs Transmission)

$$L_{xx}(\omega_i) = \frac{2n_1(\omega_i)\cos\gamma_i}{n_1(\omega_i)\cos\gamma_i + n_2(\omega_i)\cos\beta_i}$$

$$L_{yy}(\omega_i) = \frac{2n_1(\omega_i)\cos\beta_i}{n_1(\omega_i)\cos\beta_i + n_2(\omega_i)\cos\gamma_i}$$

from Hong-Fei Wang's review paper

$$L_{zz}(\omega_i) = \frac{2n_2(\omega_i)\cos\beta_i}{n_1(\omega_i)\cos\gamma_i + n_2(\omega_i)\cos\beta_i}\left(\frac{n_1(\omega_i)}{n'(\omega_i)}\right)^2$$

In[*]:=
```
RLxx = {(2 * nwaterSFG * Cos[TangleSFG Degree]) /
      (nwaterSFG * Cos[TangleSFG Degree] + nCelluloseSFG * Cos[IangleSFG Degree]),
    (2 * nwaterVis * Cos[TangleVis Degree]) / (nwaterVis * Cos[TangleVis Degree] +
       nCelluloseVis * Cos[IangleVis Degree]), (2 * nwaterIR * Cos[TangleIR Degree]) /
      (nwaterIR * Cos[TangleIR Degree] + nCelluloseIR * Cos[IangleIR Degree])};
RLyy = {(2 * nwaterSFG * Cos[IangleSFG Degree]) /
      (nwaterSFG * Cos[IangleSFG Degree] + nCelluloseSFG * Cos[TangleSFG Degree]),
    (2 * nwaterVis * Cos[IangleVis Degree]) / (nwaterVis * Cos[IangleVis Degree] +
       nCelluloseVis * Cos[TangleVis Degree]), (2 * nwaterIR * Cos[IangleIR Degree]) /
      (nwaterIR * Cos[IangleIR Degree] + nCelluloseIR * Cos[TangleIR Degree])};
RLzz = {((2 * nCelluloseSFG * Cos[IangleSFG Degree]) /
       (nwaterSFG * Cos[TangleSFG Degree] + nCelluloseSFG * Cos[IangleSFG Degree])) *
      (nwaterSFG / nCelluloseSFG)^2, ((2 * nCelluloseVis * Cos[IangleVis Degree]) /
       (nwaterVis * Cos[TangleVis Degree] + nCelluloseVis * Cos[IangleVis Degree]))
      (nwaterVis / nCelluloseVis)^2, ((2 * nCelluloseIR * Cos[IangleIR Degree]) /
       (nwaterIR * Cos[TangleIR Degree] + nCelluloseIR * Cos[IangleIR Degree]))
      (nwaterIR / nCelluloseIR)^2};


TLxx = {(2 * nCelluloseSFG * Cos[IangleSFG Degree]) /
      (nwaterSFG * Cos[TangleSFG Degree] + nCelluloseSFG * Cos[IangleSFG Degree]),
    (2 * nwaterVis * Cos[TangleVis Degree]) / (nwaterVis * Cos[TangleVis Degree] +
       nCelluloseVis * Cos[IangleVis Degree]), (2 * nwaterIR * Cos[TangleIR Degree]) /
      (nwaterIR * Cos[TangleIR Degree] + nCelluloseIR * Cos[IangleIR Degree])};
TLyy = {(2 * nCelluloseSFG * Cos[TangleSFG Degree]) /
      (nwaterSFG * Cos[IangleSFG Degree] + nCelluloseSFG * Cos[TangleSFG Degree]),
    (2 * nwaterVis * Cos[IangleVis Degree]) / (nwaterVis * Cos[IangleVis Degree] +
       nCelluloseVis * Cos[TangleVis Degree]), (2 * nwaterIR * Cos[IangleIR Degree]) /
      (nwaterIR * Cos[IangleIR Degree] + nCelluloseIR * Cos[TangleIR Degree])};
TLzz = {((2 * nwaterSFG * Cos[TangleSFG Degree]) /
       (nwaterSFG * Cos[TangleSFG Degree] + nCelluloseSFG * Cos[IangleSFG Degree])) *
      (nCelluloseSFG / nCelluloseSFG)^2, ((2 * nCelluloseVis * Cos[IangleVis Degree]) /
       (nwaterVis * Cos[TangleVis Degree] + nCelluloseVis * Cos[IangleVis Degree]))
      (nwaterVis / nCelluloseVis)^2, ((2 * nCelluloseIR * Cos[IangleIR Degree]) /
       (nwaterIR * Cos[TangleIR Degree] + nCelluloseIR * Cos[IangleIR Degree]))
      (nwaterIR / nCelluloseIR)^2};

RL = {RLxx, RLyy, RLzz}
TL = {TLxx, TLyy, TLzz}
```

Out[*]=
```
{{0.953075, 0.956256, 0.972313},
 {0.949713, 0.947076, 0.96625}, {0.86181, 0.860126, 0.908825}}
```

Out[ ]=

```
{{1.04693, 0.956256, 0.972313},
 {1.05029, 0.947076, 0.96625}, {0.953075, 0.860126, 0.908825}}
```

Interface Vectors (Reflection vs Transmission)

In[ ]:=

```
RIVSFG =
  {Sign[RangleSFG] Cos[RangleSFG Degree], 1, Sign[RangleSFG] Sin[RangleSFG Degree]} // N;
RIVVIS = {Cos[IangleVis Degree], 1, Sin[IangleVis Degree]} // N;
RIVIR =
  {Sign[IangleIR] Cos[IangleIR Degree], 1, Sign[IangleIR] Sin[IangleIR Degree]} // N;

TIVSFG =
  {Sign[TangleSFG] Cos[TangleSFG Degree], 1, Sign[TangleSFG] Sin[TangleSFG Degree]} // N;
TIVVIS = {Cos[IangleVis Degree], 1, Sin[IangleVis Degree]} // N;
TIVIR =
  {Sign[IangleIR] Cos[IangleIR Degree], 1, Sign[IangleIR] Sin[IangleIR Degree]} // N;

RIV = {RIVSFG, RIVVIS, RIVIR}
TIV = {TIVSFG, TIVVIS, TIVIR}
```

Out[ ]=

```
{{-0.981409, 1., 0.191928}, {0.951057, 1., 0.309017}, {-0.951057, 1., 0.309017}}
```

Out[ ]=

```
{{0.984722, 1., 0.174135}, {0.951057, 1., 0.309017}, {-0.951057, 1., 0.309017}}
```

Orientation Matrix Definition (Euler + polar coordinate)

eulphi, eultheta, eulpsi = Euler angles
dipole = α (with repect to chain axis),
β (with repect to the normal of chain axis)
temp1 = Sum of the X component and the Z component in Parallel packing
temp2 = Sum of the X component and the Z component in Antiparallel packing
pp = parallel packing
ap = antiparallel packing

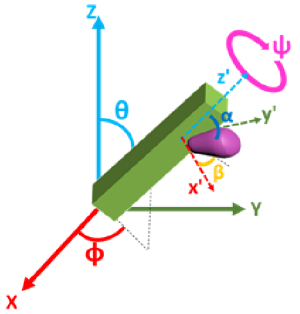

Rotation order has to be chosen to explain the rotational system.
Xing Chen’s DFG Work : z-x-z rotation
This Work : z-y-z rotation (why? to make the fiber rotate in laser incidence plane (xz plane) when ϕ is zero)

eul : Euler Rotation Matrix
dipole : Molecular dipole

$$\begin{pmatrix} \begin{pmatrix} \mathrm{Cos}[b]\,\mathrm{Sin}[\alpha]\,\beta_{x,x,x} \\ \mathrm{Sin}[b]\,\mathrm{Sin}[\alpha]\,\beta_{x,x,y} \\ \mathrm{Cos}[\alpha]\,\beta_{x,x,z} \end{pmatrix} & \begin{pmatrix} \mathrm{Cos}[b]\,\mathrm{Sin}[\alpha]\,\beta_{x,y,x} \\ \mathrm{Sin}[b]\,\mathrm{Sin}[\alpha]\,\beta_{x,y,y} \\ \mathrm{Cos}[\alpha]\,\beta_{x,y,z} \end{pmatrix} & \begin{pmatrix} \mathrm{Cos}[b]\,\mathrm{Sin}[\alpha]\,\beta_{x,z,x} \\ \mathrm{Sin}[b]\,\mathrm{Sin}[\alpha]\,\beta_{x,z,y} \\ \mathrm{Cos}[\alpha]\,\beta_{x,z,z} \end{pmatrix} \\ \begin{pmatrix} \mathrm{Cos}[b]\,\mathrm{Sin}[\alpha]\,\beta_{y,x,x} \\ \mathrm{Sin}[b]\,\mathrm{Sin}[\alpha]\,\beta_{y,x,y} \\ \mathrm{Cos}[\alpha]\,\beta_{y,x,z} \end{pmatrix} & \begin{pmatrix} \mathrm{Cos}[b]\,\mathrm{Sin}[\alpha]\,\beta_{y,y,x} \\ \mathrm{Sin}[b]\,\mathrm{Sin}[\alpha]\,\beta_{y,y,y} \\ \mathrm{Cos}[\alpha]\,\beta_{y,y,z} \end{pmatrix} & \begin{pmatrix} \mathrm{Cos}[b]\,\mathrm{Sin}[\alpha]\,\beta_{y,z,x} \\ \mathrm{Sin}[b]\,\mathrm{Sin}[\alpha]\,\beta_{y,z,y} \\ \mathrm{Cos}[\alpha]\,\beta_{y,z,z} \end{pmatrix} \\ \begin{pmatrix} \mathrm{Cos}[b]\,\mathrm{Sin}[\alpha]\,\beta_{z,x,x} \\ \mathrm{Sin}[b]\,\mathrm{Sin}[\alpha]\,\beta_{z,x,y} \\ \mathrm{Cos}[\alpha]\,\beta_{z,x,z} \end{pmatrix} & \begin{pmatrix} \mathrm{Cos}[b]\,\mathrm{Sin}[\alpha]\,\beta_{z,y,x} \\ \mathrm{Sin}[b]\,\mathrm{Sin}[\alpha]\,\beta_{z,y,y} \\ \mathrm{Cos}[\alpha]\,\beta_{z,y,z} \end{pmatrix} & \begin{pmatrix} \mathrm{Cos}[b]\,\mathrm{Sin}[\alpha]\,\beta_{z,z,x} \\ \mathrm{Sin}[b]\,\mathrm{Sin}[\alpha]\,\beta_{z,z,y} \\ \mathrm{Cos}[\alpha]\,\beta_{z,z,z} \end{pmatrix} \end{pmatrix}$$

In[ ]:=
```
(*Orientation Definition*)

eul = EulerMatrix[{ϕ, θ, ψ}, {3, 2, 3}];
dipole = {Sin[α] Cos[b], Sin[α] Sin[b], Cos[α]};
(*hyper=Table[dipole[[k]]*β_{i ,j ,k}, {i,1,3},{j,1,3},{k,1,3}] /. {1→x,2→y,3→z};*)
(*hyperOH=Table[dipole[[k]]*β_{i ,j ,k}, {i,1,3},{j,1,3},{k,1,3}]/. {1→x,2→y,3→z};
(*we assume that all β is √2.2 from Amin's 2019 JPC experimental data*)
hyperCH=Table[dipole[[k]]*β_{i ,j ,k}, {i,1,3},{j,1,3},{k,1,3}]/. {1→x,2→y,3→z};
(*we assume that all β is 1*)*)

hyperOH = Table[dipole[[k]] * 0.9, {i, 1, 3}, {j, 1, 3}, {k, 1, 3}];
(*we assume that all β is √2.2 from Amin's 2019 JPC experimental data*)
hyperCH = Table[dipole[[k]] * 1, {i, 1, 3}, {j, 1, 3}, {k, 1, 3}];
(*we assume that all β is 1*)

Rchai2OH[var1_, var2_, var3_] :=
 Sum_{i=1}^{3} Sum_{j=1}^{3} Sum_{k=1}^{3} (eul[[var1]][[i]] * eul[[var2]][[j]] * eul[[var3]][[k]] * RL[[var1, 1]] * RL[[var2, 2]] *
    RL[[var3, 3]] * RIV[[1, var1]] * RIV[[2, var2]] * RIV[[3, var3]] * hyperOH[[i]][[j]][[k]])
Rchai2CH[var1_, var2_, var3_] :=
 Sum_{i=1}^{3} Sum_{j=1}^{3} Sum_{k=1}^{3} (eul[[var1]][[i]] * eul[[var2]][[j]] * eul[[var3]][[k]] * RL[[var1, 1]] * RL[[var2, 2]] *
    RL[[var3, 3]] * RIV[[1, var1]] * RIV[[2, var2]] * RIV[[3, var3]] * hyperCH[[i]][[j]][[k]])

Tchai2OH[var1_, var2_, var3_] :=
 Sum_{i=1}^{3} Sum_{j=1}^{3} Sum_{k=1}^{3} (eul[[var1]][[i]] * eul[[var2]][[j]] * eul[[var3]][[k]] * TL[[var1, 1]] * TL[[var2, 2]] *
    TL[[var3, 3]] * TIV[[1, var1]] * TIV[[2, var2]] * TIV[[3, var3]] * hyperOH[[i]][[j]][[k]])
Tchai2CH[var1_, var2_, var3_] :=
 Sum_{i=1}^{3} Sum_{j=1}^{3} Sum_{k=1}^{3} (eul[[var1]][[i]] * eul[[var2]][[j]] * eul[[var3]][[k]] * TL[[var1, 1]] * TL[[var2, 2]] *
    TL[[var3, 3]] * TIV[[1, var1]] * TIV[[2, var2]] * TIV[[3, var3]] * hyperCH[[i]][[j]][[k]])
```

Polarization Combination (Reflection, Transmission -> Sign)

PPP xxx/zxx/xzx/xxz /xzz/zxz/zzx/ zzz

PSP xyx/zyx/xyz/zyz

SPP yxx/yzx/yxz/yzz

SSP yyx/yyz
SSS yyy
PSS xyy/zyy
PPS xxy/zxy/xzy/zzy
SPS yxy/yzy

```
(*reflection*)

OHdipole = {α → 30 Degree, b → 0 Degree};
CHdipole = {α → 60 Degree, b → 90 Degree};

RSSPOH = (Rchai2OH[2, 2, 1] + Rchai2OH[2, 2, 3]) /. OHdipole // Simplify;
RSSPCH = (Rchai2CH[2, 2, 1] + Rchai2CH[2, 2, 3]) /. CHdipole // Simplify;

RPPPOH = ( Rchai2OH[1, 1, 1] + Rchai2OH[3, 1, 1] +
      Rchai2OH[1, 3, 1] + Rchai2OH[1, 1, 3] + Rchai2OH[1, 3, 3] + Rchai2OH[3, 1, 3] +
      Rchai2OH[3, 3, 1] + Rchai2OH[3, 3, 3]) /. OHdipole // Simplify;
RPPPCH = ( Rchai2CH[1, 1, 1] + Rchai2CH[3, 1, 1] +
      Rchai2CH[1, 3, 1] + Rchai2CH[1, 1, 3] + Rchai2CH[1, 3, 3] + Rchai2CH[3, 1, 3] +
      Rchai2CH[3, 3, 1] + Rchai2CH[3, 3, 3]) /. CHdipole // Simplify;

RPSPOH = ( Rchai2OH[1, 2, 1] + Rchai2OH[3, 2, 1] + Rchai2OH[1, 2, 3] + Rchai2OH[3, 2, 3]) /.
    OHdipole // Simplify;
RPSPCH = ( Rchai2CH[1, 2, 1] + Rchai2CH[3, 2, 1] + Rchai2CH[1, 2, 3] + Rchai2CH[3, 2, 3]) /.
    CHdipole // Simplify;

RSPPOH = ( Rchai2OH[2, 1, 1] + Rchai2OH[2, 3, 1] + Rchai2OH[2, 1, 3] + Rchai2OH[2, 3, 3]) /.
    OHdipole // Simplify;
RSPPCH = ( Rchai2CH[2, 1, 1] + Rchai2CH[2, 3, 1] + Rchai2CH[2, 1, 3] + Rchai2CH[2, 3, 3]) /.
    CHdipole // Simplify;

RSSSOH = ( Rchai2OH[2, 2, 2]) /. OHdipole // Simplify;
RSSSCH = ( Rchai2CH[2, 2, 2]) /. CHdipole // Simplify;

RPSSOH = (Rchai2OH[1, 2, 2] + Rchai2OH[3, 2, 2]) /. OHdipole // Simplify;
RPSSCH = (Rchai2CH[1, 2, 2] + Rchai2CH[3, 2, 2]) /. CHdipole // Simplify;

RPPSOH = (Rchai2OH[1, 1, 2] + Rchai2OH[3, 1, 2] + Rchai2OH[1, 3, 2] + Rchai2OH[3, 3, 2]) /.
    OHdipole // Simplify;
RPPSCH = (Rchai2CH[1, 1, 2] + Rchai2CH[3, 1, 2] + Rchai2CH[1, 3, 2] + Rchai2CH[3, 3, 2]) /.
    CHdipole // Simplify;

RSPSOH = (Rchai2OH[2, 1, 2] + Rchai2OH[2, 3, 2]) /. OHdipole // Simplify;
RSPSCH = (Rchai2CH[2, 1, 2] + Rchai2CH[2, 3, 2]) /. CHdipole // Simplify;

(*Tranmission*)
TSSPOH = (Tchai2OH[2, 2, 1] + Tchai2OH[2, 2, 3]) /. OHdipole // Simplify;
```

Printed by Wolfram Mathematica Student Edition

```
TSSPCH = (Tchai2CH[2, 2, 1] + Tchai2CH[2, 2, 3]) /. CHdipole // Simplify;

TPPPOH = ( Tchai2OH[1, 1, 1] + Tchai2OH[3, 1, 1] +
      Tchai2OH[1, 3, 1] + Tchai2OH[1, 1, 3] + Tchai2OH[1, 3, 3] + Tchai2OH[3, 1, 3] +
      Tchai2OH[3, 3, 1] + Tchai2OH[3, 3, 3]) /. OHdipole // Simplify;
TPPPCH = ( Tchai2CH[1, 1, 1] + Tchai2CH[3, 1, 1] +
      Tchai2CH[1, 3, 1] + Tchai2CH[1, 1, 3] + Tchai2CH[1, 3, 3] + Tchai2CH[3, 1, 3] +
      Tchai2CH[3, 3, 1] + Tchai2CH[3, 3, 3]) /. CHdipole // Simplify;

TPSPOH = ( Tchai2OH[1, 2, 1] + Tchai2OH[3, 2, 1] + Tchai2OH[1, 2, 3] + Tchai2OH[3, 2, 3]) /.
    OHdipole // Simplify;
TPSPCH = ( Tchai2CH[1, 2, 1] + Tchai2CH[3, 2, 1] + Tchai2CH[1, 2, 3] + Tchai2CH[3, 2, 3]) /.
    CHdipole // Simplify;

TSPPOH = ( Tchai2OH[2, 1, 1] + Tchai2OH[2, 3, 1] + Tchai2OH[2, 1, 3] + Tchai2OH[2, 3, 3]) /.
    OHdipole // Simplify;
TSPPCH = ( Tchai2CH[2, 1, 1] + Tchai2CH[2, 3, 1] + Tchai2CH[2, 1, 3] + Tchai2CH[2, 3, 3]) /.
    CHdipole // Simplify;

TSSSOH = ( Tchai2OH[2, 2, 2]) /. OHdipole // Simplify;
TSSSCH = ( Tchai2CH[2, 2, 2]) /. CHdipole // Simplify;

TPSSOH = (Tchai2OH[1, 2, 2] + Tchai2OH[3, 2, 2]) /. OHdipole // Simplify;
TPSSCH = (Tchai2CH[1, 2, 2] + Tchai2CH[3, 2, 2]) /. CHdipole // Simplify;

TPPSOH = (Tchai2OH[1, 1, 2] + Tchai2OH[3, 1, 2] + Tchai2OH[1, 3, 2] + Tchai2OH[3, 3, 2]) /.
    OHdipole // Simplify;
TPPSCH = (Tchai2CH[1, 1, 2] + Tchai2CH[3, 1, 2] + Tchai2CH[1, 3, 2] + Tchai2CH[3, 3, 2]) /.
    CHdipole // Simplify;

TSPSOH = (Tchai2OH[2, 1, 2] + Tchai2OH[2, 3, 2]) /. OHdipole // Simplify;
TSPSCH = (Tchai2CH[2, 1, 2] + Tchai2CH[2, 3, 2]) /. CHdipole // Simplify;
```

Mode : Reflection or Transmission || mode= (1 Reflection), (2 Transmission)

dE = Directional Excess %/100 || dE={0, 0.5, 1.0}

θanlgles = Tilt angle || θanlgles ={0,45,90} or θanlgles=Table[i,{i,0,90,45}]

sD : Standard Deviation

u= Diameter

dz = Distance

Lc = Coherence Length

```
mode = 2;
dE = {0, 0.25, 0.5, 0.75, 1.0};
θanlgles = {0, 10, 20};
sD = {10};
u = 4;
dz = 1;
Lc = 50 * 10^-9;
```

```
pc = 1;
While[pc ≤ 8,
 Piecewise[{{
    polarizationcombination = {RSSP, RPPS, RPPP, RSSS, RPSP, RSPS, RSPP, RPSS}, mode == 1}, {
    polarizationcombination = {TSSP, TPPS, TPPP, TSSS, TPSP, TSPS, TSPP, TPSS}, mode == 2}}];

 pcOH = ToExpression[ToString[polarizationcombination[[pc]]] <> "OH"];
 pcCH = ToExpression[ToString[polarizationcombination[[pc]]] <> "CH"];
 Piecewise[{{dk = dkr, mode == 1}, {dk = dkt, mode == 2}}];


 dEN = 1;
 dEList = dE;

 While[dEN < Length[dEList] + 1,

  θcasesN = 1;
  θcases = θanlgles;

  While[θcasesN ≤ Length[θcases],

   θfix = θcases[[θcasesN]];

   sDN = 1;
   sDList = sD;
   While[sDN ≤ Length[sDList],

    ϕtemp = 0;
    temp3 = {};

    While[ϕtemp < 365,

     θ1 = θfix Degree;
     ϕ1 = ϕtemp Degree;
```

```
Ori1 := {ϕ → RandomVariate[NormalDistribution[ϕ1, 10 Degree]],
  θ → RandomVariate[NormalDistribution[θ1, 10 Degree]], ψ → RandomReal[{0, 2 Pi}]};

INTERATION = 5000;

Lcc = Lc * 10^9;
m = Lc / (u * 10^-9 + dz * 10^-9);

G = Table[0, {w, 1, INTERATION}];
J = Table[0, {w, 1, INTERATION}];
order = Table[0, {w, 1, INTERATION}];
ddd = Table[{0, 0, 0}, {w, Lcc}];
fff = Table[{0, 0, 0}, {w, Lcc}];

p = 1;
q = 1;

ddd[[(p - 1) Lcc + q, 2]] = u;
ddd[[(p - 1) Lcc + q, 1]] = dz;

fff[[(p - 1) Lcc + q, 2]] = u;
fff[[(p - 1) Lcc + q, 1]] = dz;


For[w = 1, w < INTERATION + 1, w++,
 detemp1 = RandomVariate[
   TruncatedDistribution[{-1, 1}, NormalDistribution[dEList[[dEN]], 0.2]]];
 seqtemp = Flatten[{Table[1, {i, 1, Round[m * (detemp1 + 1) / 2]}],
    Table[0, {i, 1, Round[m * (1 - (detemp1 + 1) / 2)]}]}];
 seq = RandomSample[seqtemp];

 OHtemp1 = Table[0, {k, 0, m - 1}];
 CHtemp1 = Table[0, {k, 0, m - 1}];


 For[i = 0, i ≤ (m - 1), i++,
  Oritemp1 = Ori1;
  Oritemp2 = {ϕ → Oritemp1[[1]][[2]], θ → Oritemp1[[2]][[2]] + Pi, ψ → Oritemp1[[3]][[2]] + Pi};

  Oritemp3 = If[seq[[i + 1]] == 1, Oritemp1, Oritemp2];

  OHtemp2 =
   ((Exp[-I dk u * 10^-9] - 1) / dk) (pcOH /. Oritemp3) Exp[- I dk i (u * 10^-9 + dz * 10^-9)];
  CHtemp2 =
   ((Exp[-I dk u * 10^-9] - 1) / dk) (pcCH /. Oritemp3) Exp[- I dk i (u * 10^-9 + dz * 10^-9)];
```

```
          OHtemp1[[i + 1]] = OHtemp2;
          CHtemp1[[i + 1]] = CHtemp2;

         ];

         tempOH = Total[OHtemp1];
         tempCH = Total[CHtemp1];

         G[[w]] = (Conjugate[tempOH] * tempOH) * (1 / Cos[TangleSFG Degree])^2;
         J[[w]] = (Conjugate[tempCH] * tempCH) * (1 / Cos[TangleSFG Degree])^2;

         order[[w]] = Count[seqtemp, 1];

        ];

        ddd[[(p - 1) Lcc + q, 3]] = Mean[G];
        fff[[(p - 1) Lcc + q, 3]] = Mean[J];

        OHCHpara = Table[{ϕtemp, θfix, θfix, ddd[[i, 1]],
           ddd[[i, 2]], If [Re[fff[[i, 3]]] > 0, Re[ddd[[i, 3]]] / Re[fff[[i, 3]]], 0],
           Re[ddd[[i, 3]]], Re[fff[[i, 3]]]}, {i, 1, Length[ddd]}];
        temp3 = AppendTo[temp3, OHCHpara[[1]]];

        ϕtemp = ϕtemp + 15;];

       Export[NotebookDirectory[] <> "DE" <> ToString[dEList[[dEN]] * 100] <>
         " -PPS-Azimuthal-T-(m10)-(" <> ToString[θ1] <> ").csv", temp3];

       sDN = sDN + 1;

      ];

      θcasesN = θcasesN + 1;

     ];

     dEN = dEN + 1;
    ];

    pc = pc + 1;
   ]
```
