# Supplementary material for: Tissue-specific directionality of cellulose synthase complex movement inferred from cellulose microfibril polarity in secondary cell walls of Arabidopsis
Source: Sci Rep. 2023 Dec 12;13:22007. doi: 10.1038/s41598-023-48545-z (PMC10716418; doi:10.1038/s41598-023-48545-z)
Supplement: Supplementary file 3 — Supplementary Figures. [file 41598_2023_48545_MOESM3_ESM.docx]

Supplementary information for

Tissue-Specific Directionality of Cellulose Synthase Complex Movement Inferred from Cellulose Microfibril Polarity in Secondary Cell Walls of Arabidopsis

Juseok Choi^1+^, Mohamadamin Makarem^1+^, Chonghan Lee,^2^ Jongcheol Lee,^1^ Sarah Kiemle,^3^ Daniel J. Cosgrove,^4^ and Seong H. Kim^1*^

^1^ Department of Chemical Engineering, Materials Research Institute, Pennsylvania State University, University Park, Pennsylvania 16802, USA.

^2^ Department of Computer Science and Engineering, Pennsylvania State University, University Park, Pennsylvania 16802, USA.

^3^ Materials Characterization Laboratory, Pennsylvania State University, University Park, Pennsylvania 16802, USA.

^4^ Department of Biology, Pennsylvania State University, University Park, Pennsylvania 16802, USA.

+ These authors contributed equally to this work

* Corresponding author: [shk10@psu.edu](mailto:shk10@psu.edu)

**
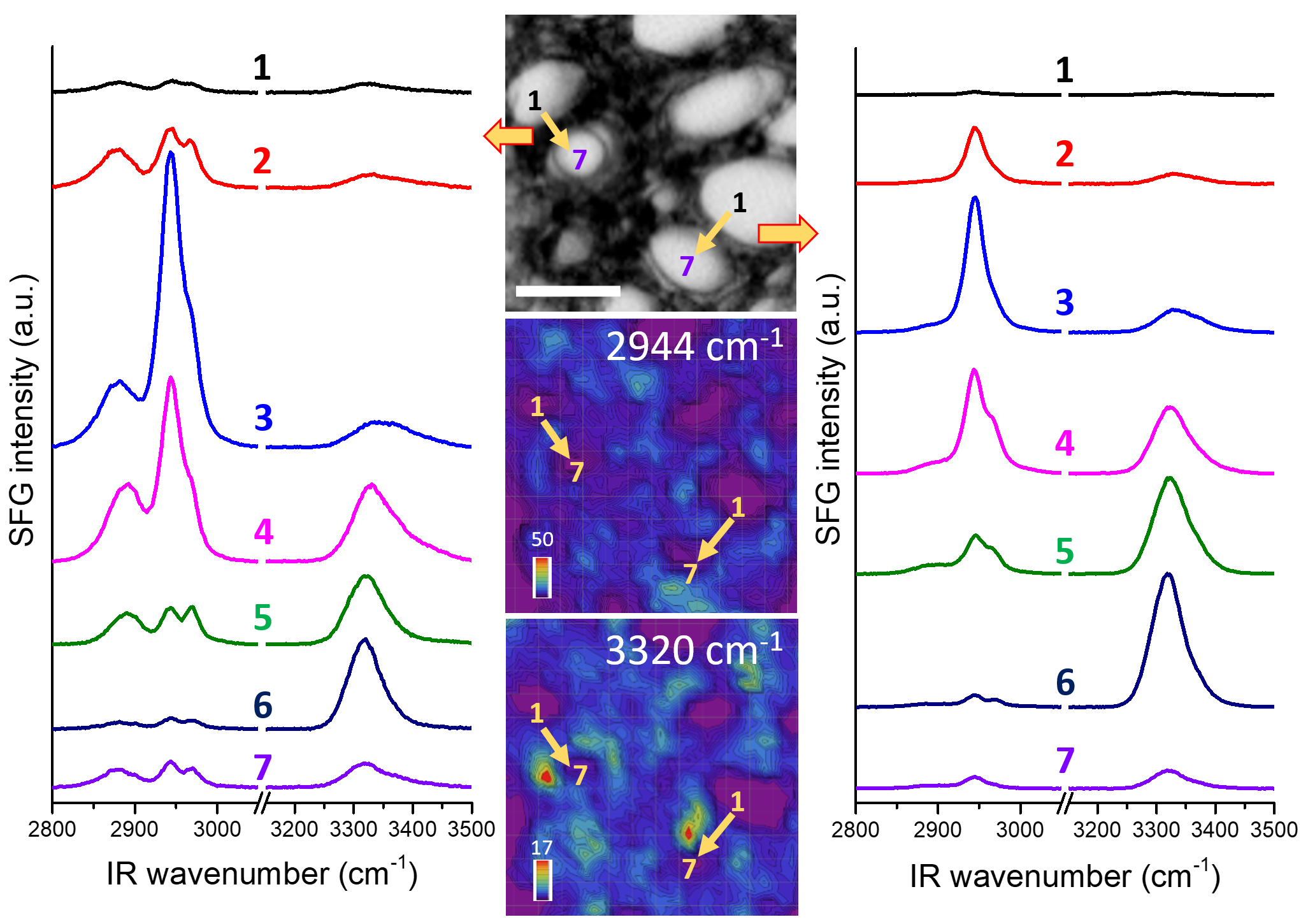
**

**Supplementary** **Figure 1.** SFG spectra extracted from seven locations across two xylem cell walls touching each other. The spectra are extracted along the arrow directions (numbered from 1 through 7 every 2 μm) at two locations from the hyperspectral SFG images.


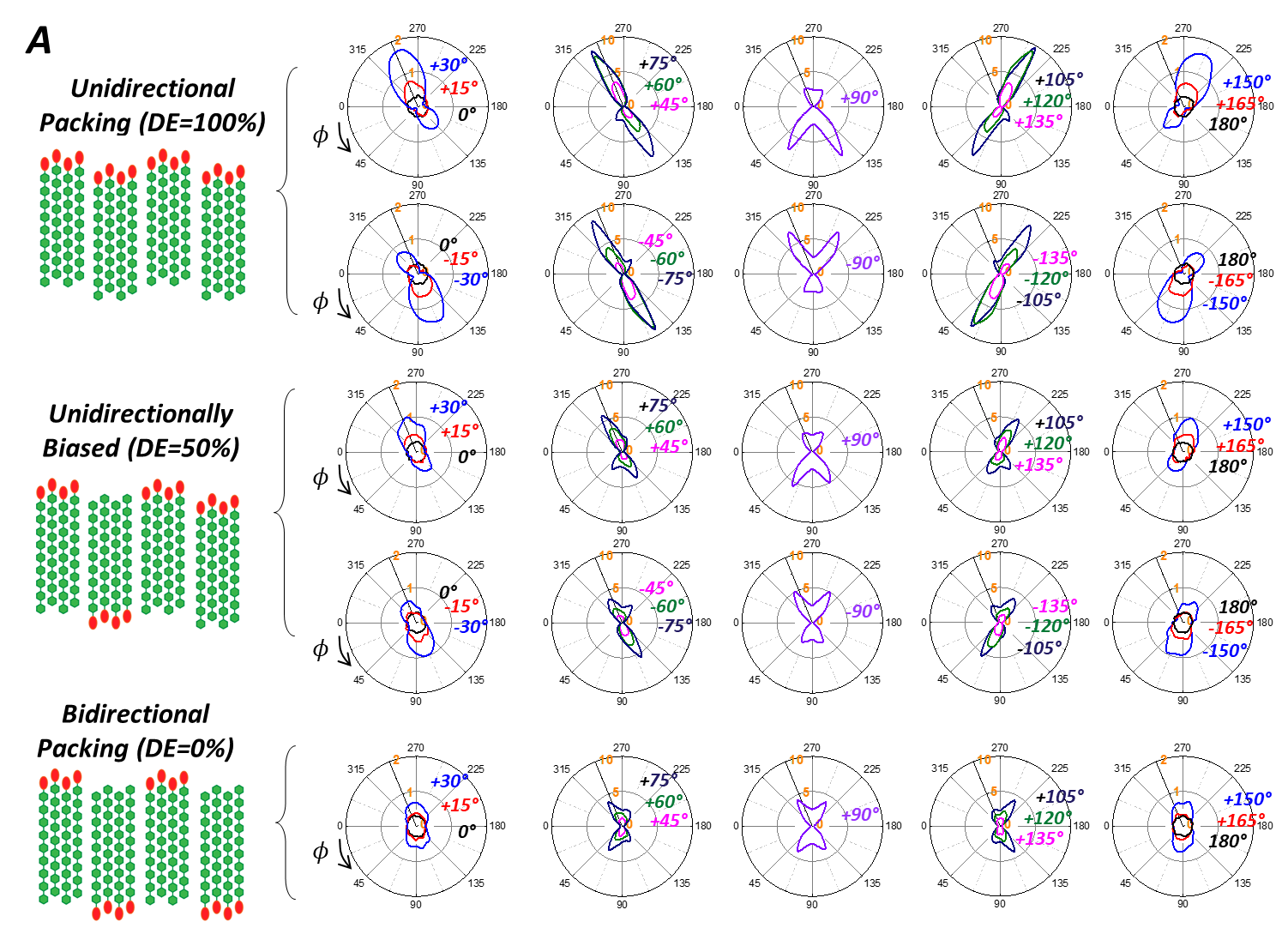


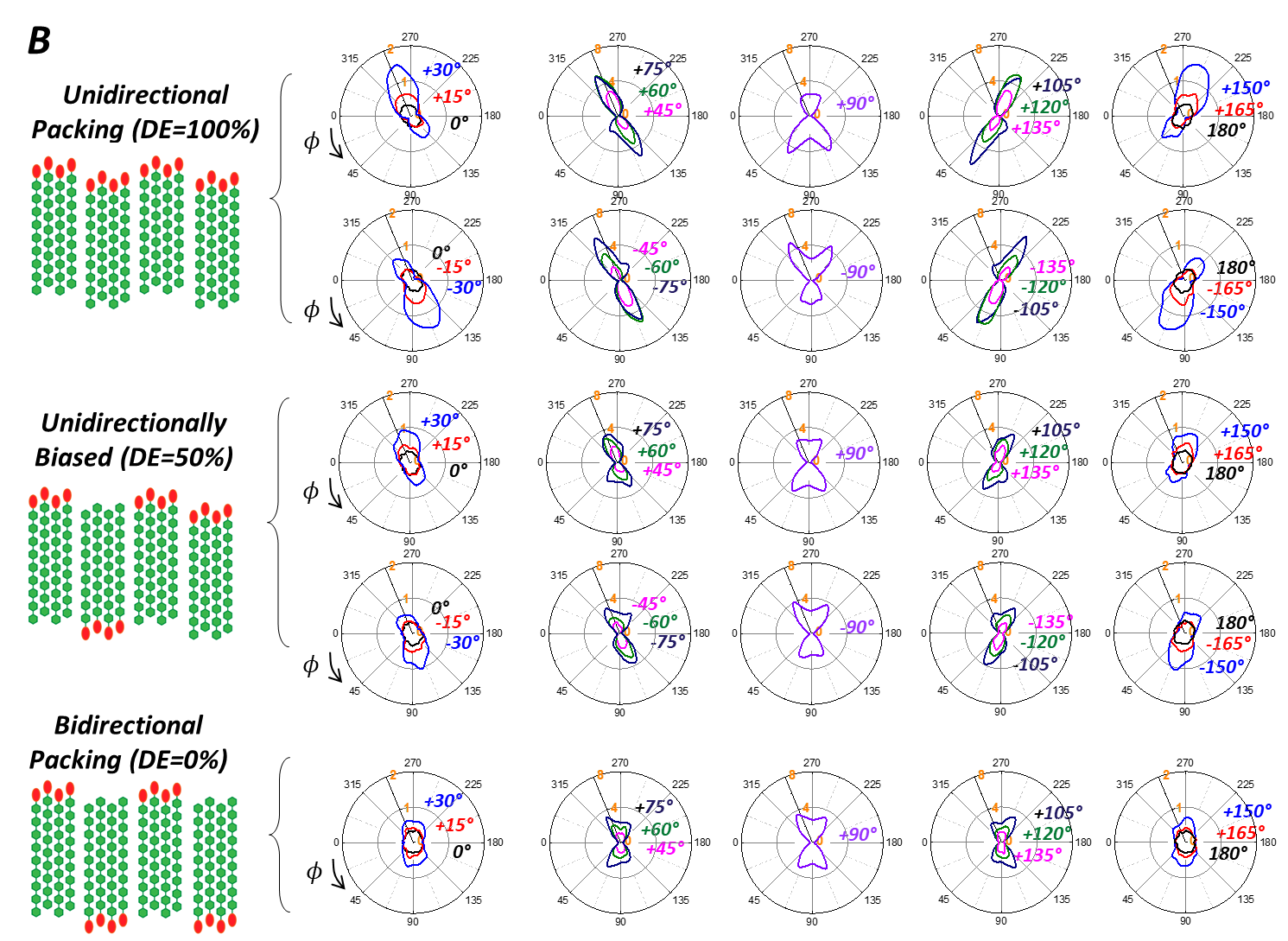


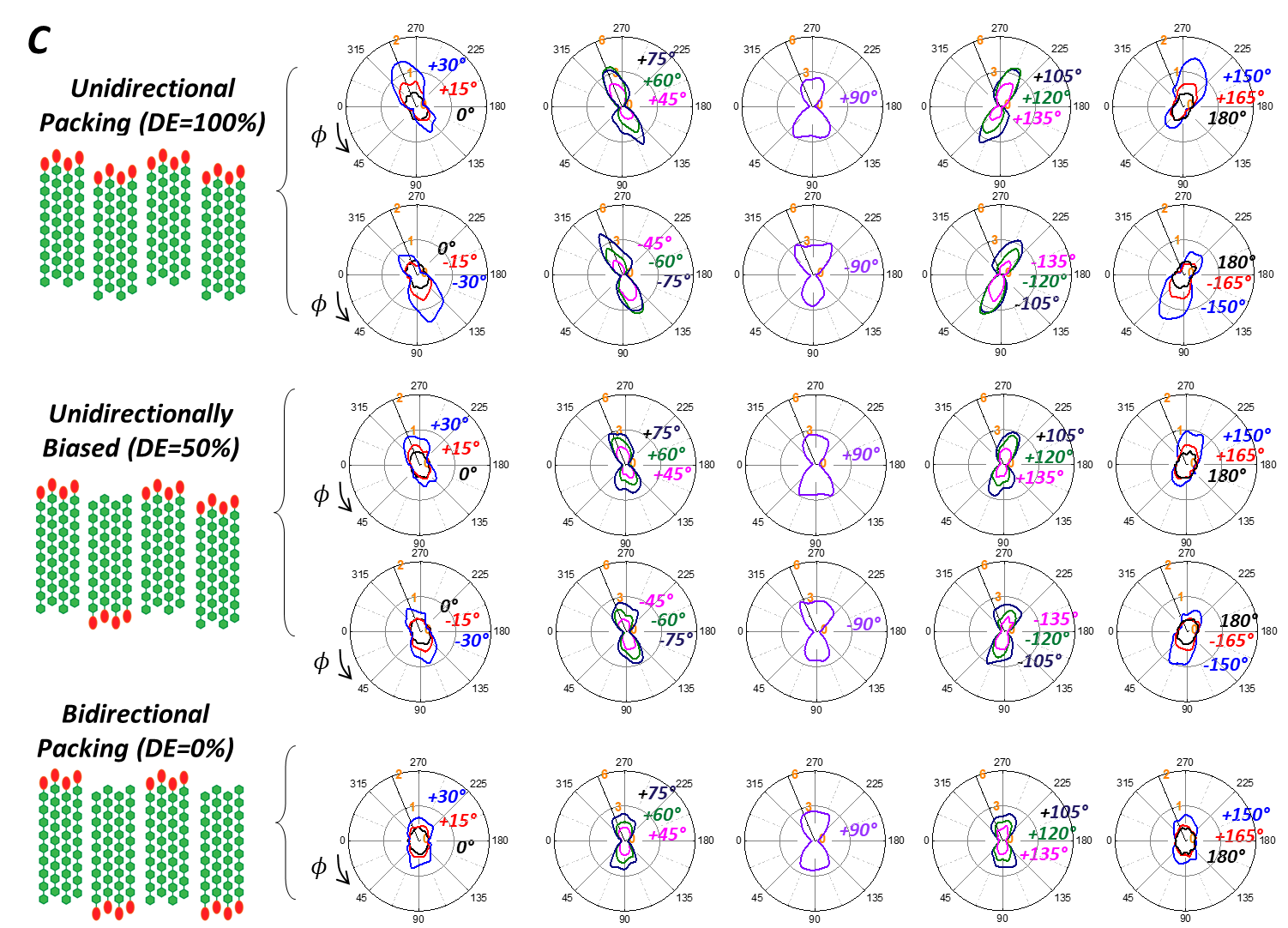


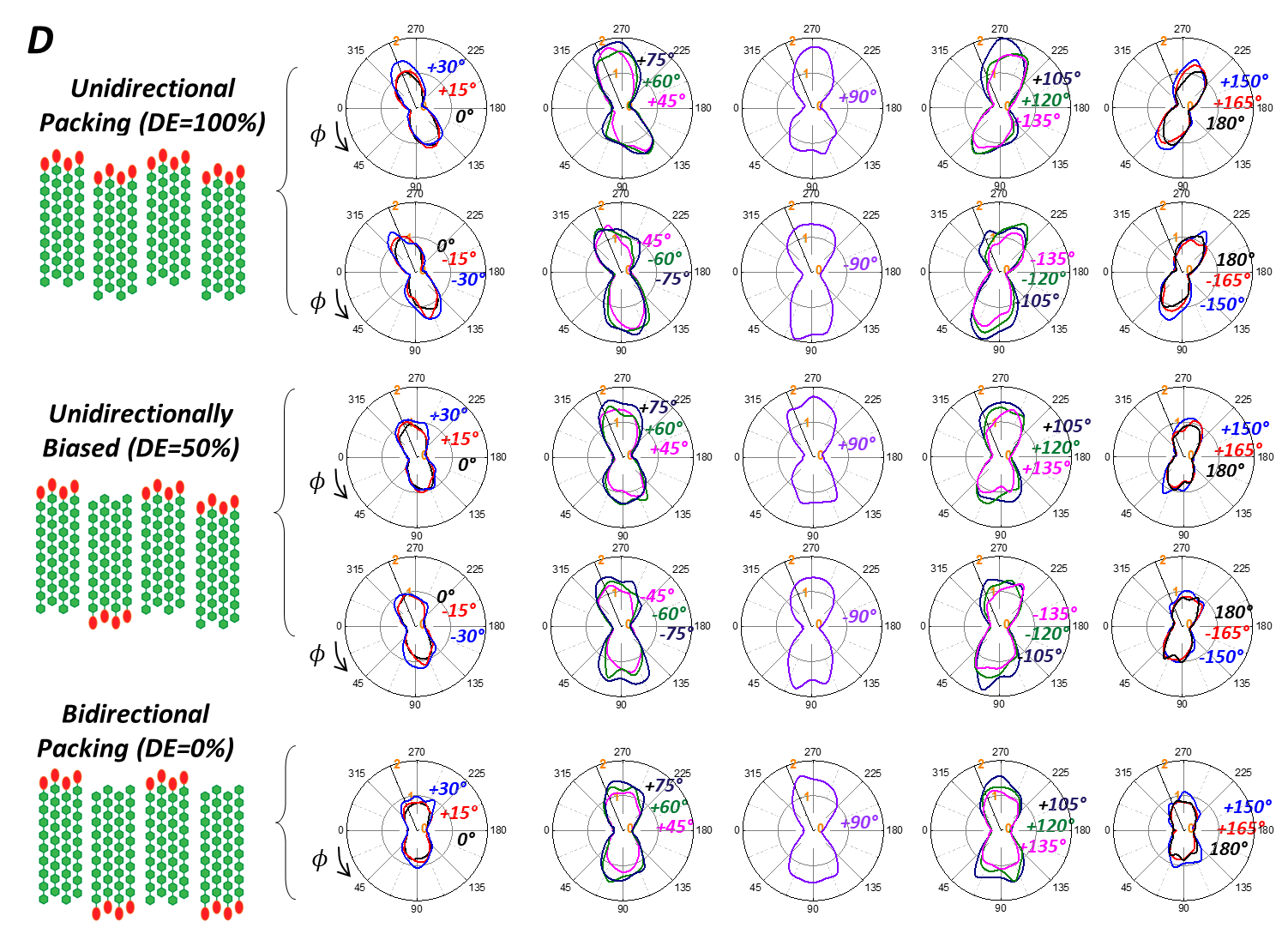


**Supplementary** **Figure 2.** Polar plots of the 3320cm^-1^/2944cm^-1^ *pps-*SFG intensity ratio (OH/CH) as a function of azimuth angle (φ) at selected tilt angle (θ; shown as numbers in each plot) for unidirectional (*DE* = 100%), partially unidirectional (*DE* = 50%), and bidirectional (*DE* = 0%) packing of CMFs with different σ_φ_ and σ_θ_; (A) σ_φ_ = σ_θ_ = 5°, (B) σ_φ_ = σ_θ_ = 10°, (C) σ_φ_ = σ_θ_ = 15°, (D) σ_φ_ = 10, σ_θ_ = 45°. The other conditions are set to the same values in Fig. 2.


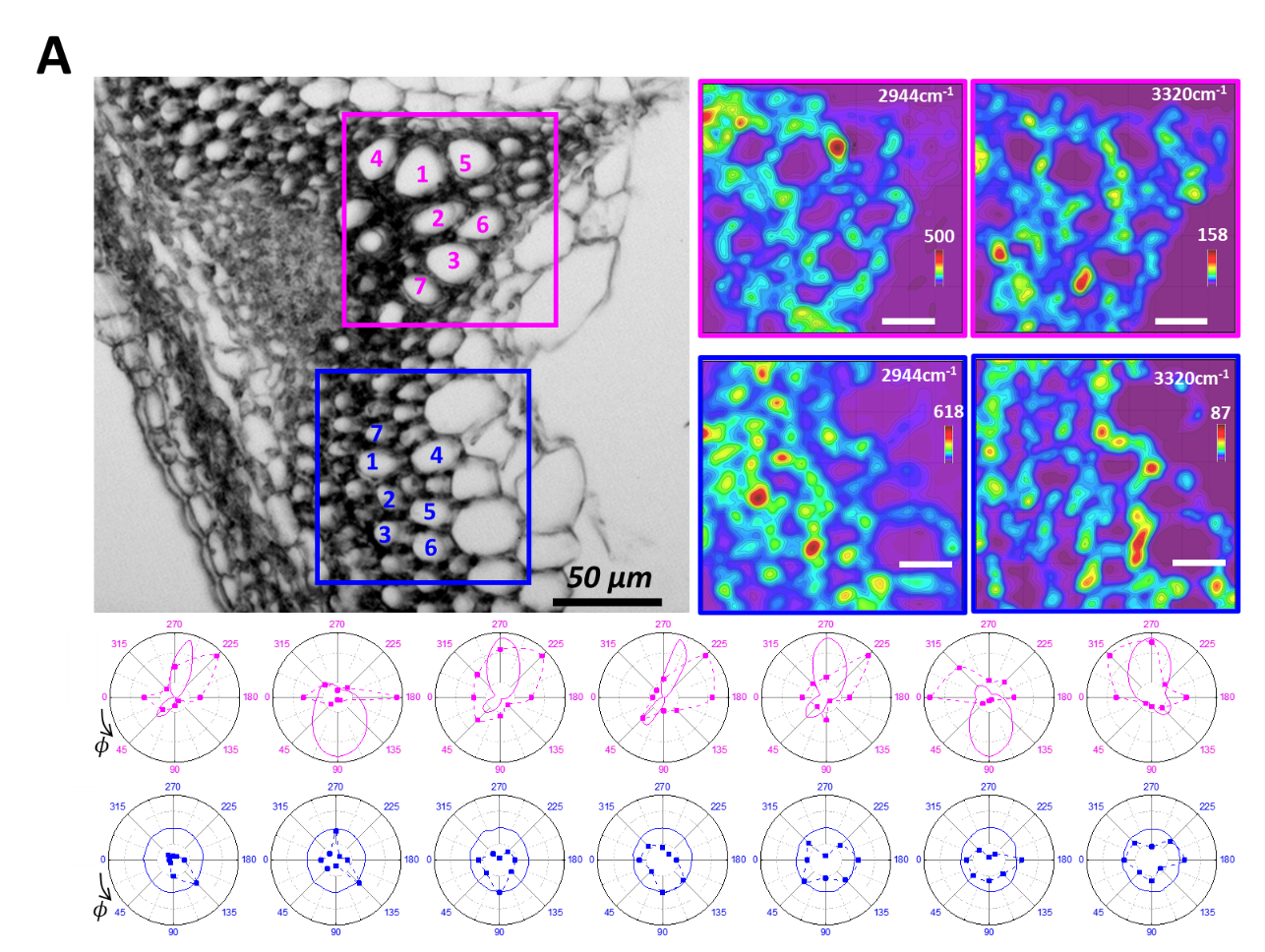


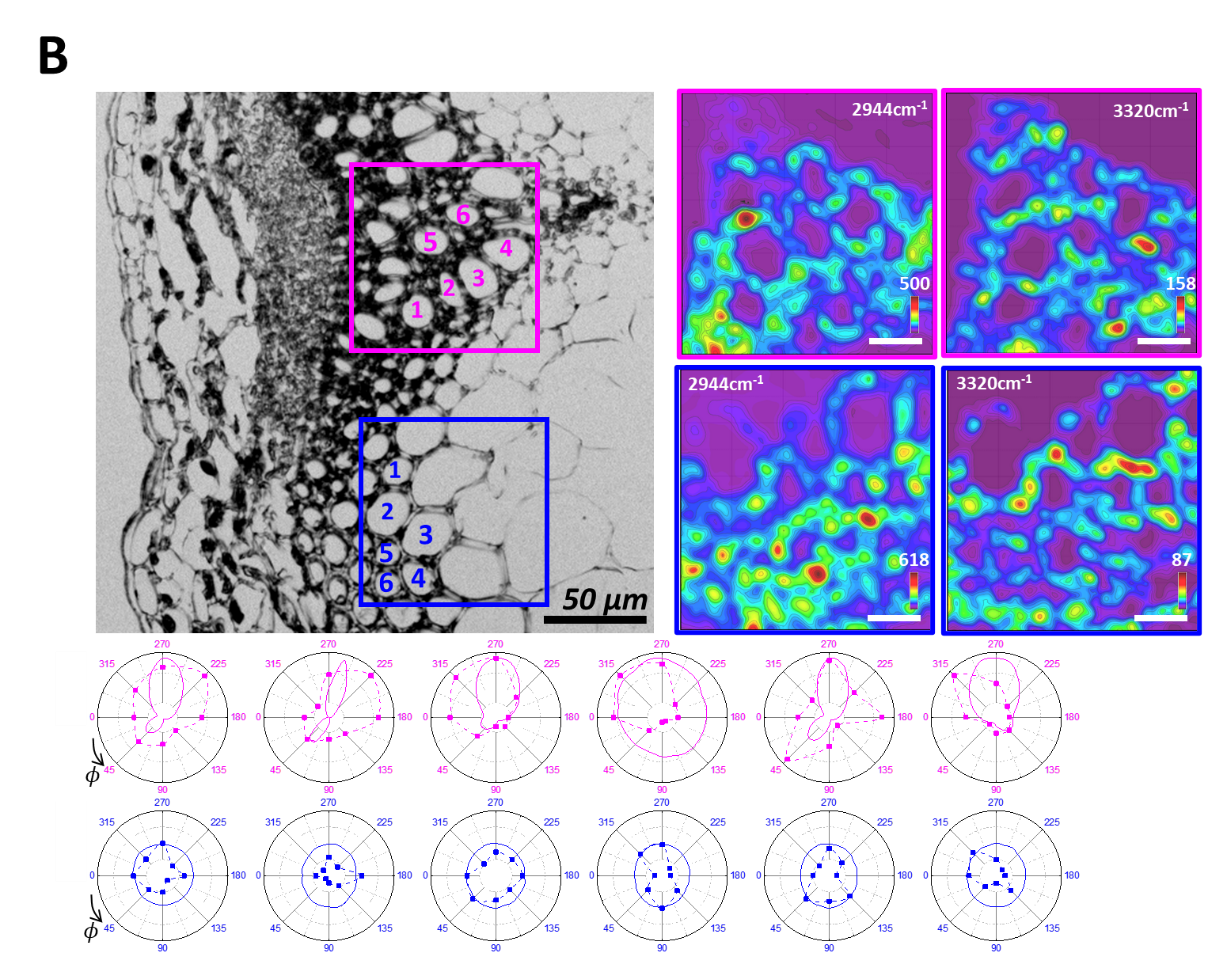


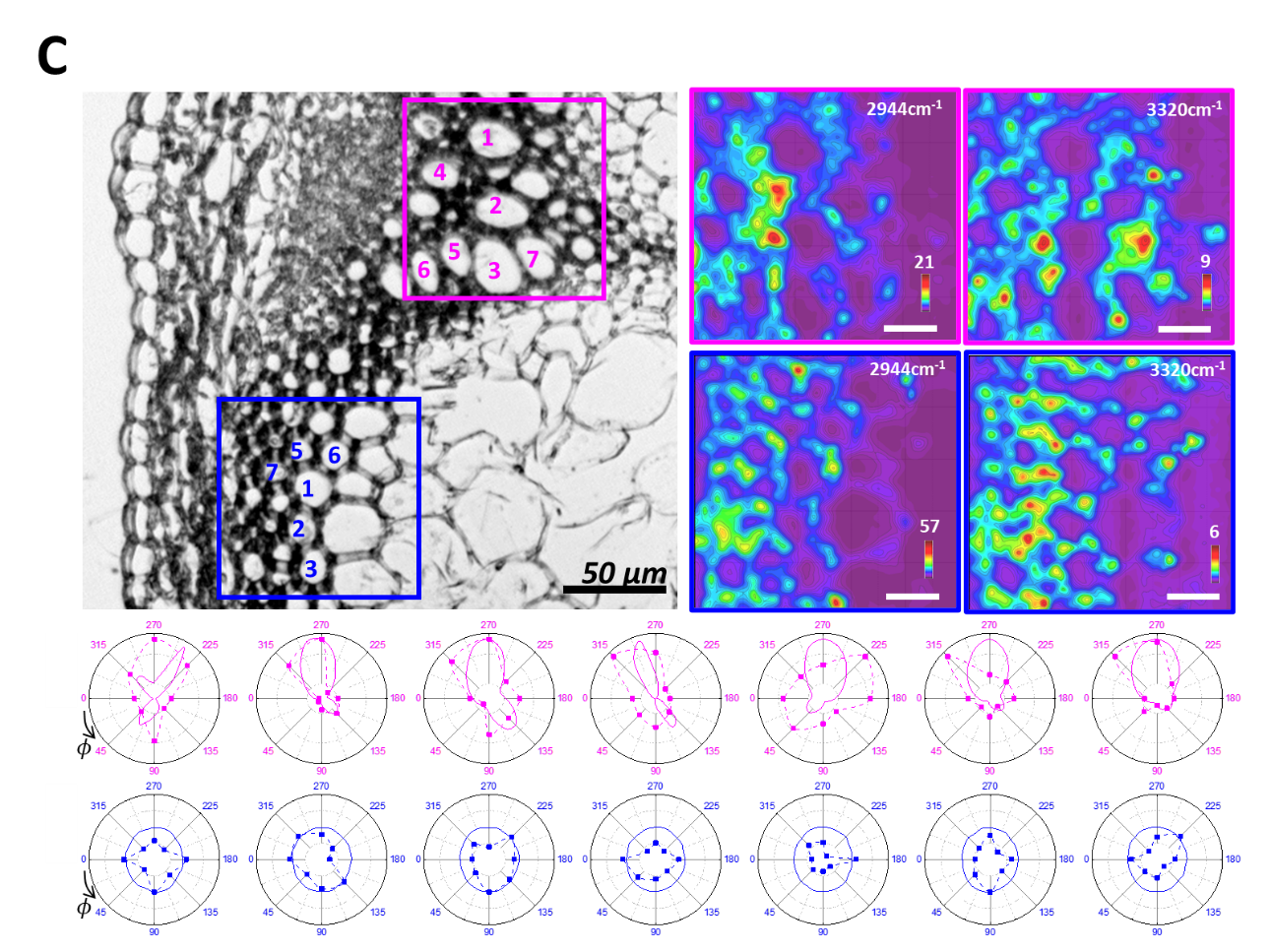


Supplementary Figure 3. Hyperspectral intensity maps of 2944 cm^-1^ and 3320 cm^-1^ are shown for the xylem and IFF regions marked with pink and blue boxes, respectively, in the optical images of three cross-sectioned samples of Arabidopsis stems (A, B, and C). The polar plots right below images show the experimentally-generated (dotted line) intensity ratio of the 3320 cm^-1^ OH peak versus the 2944 cm^-1^ CH peak at 8 locations around one single cell. The numerically calculated intensity ratio based on MLP analysis (solid line) are plotted together.

**
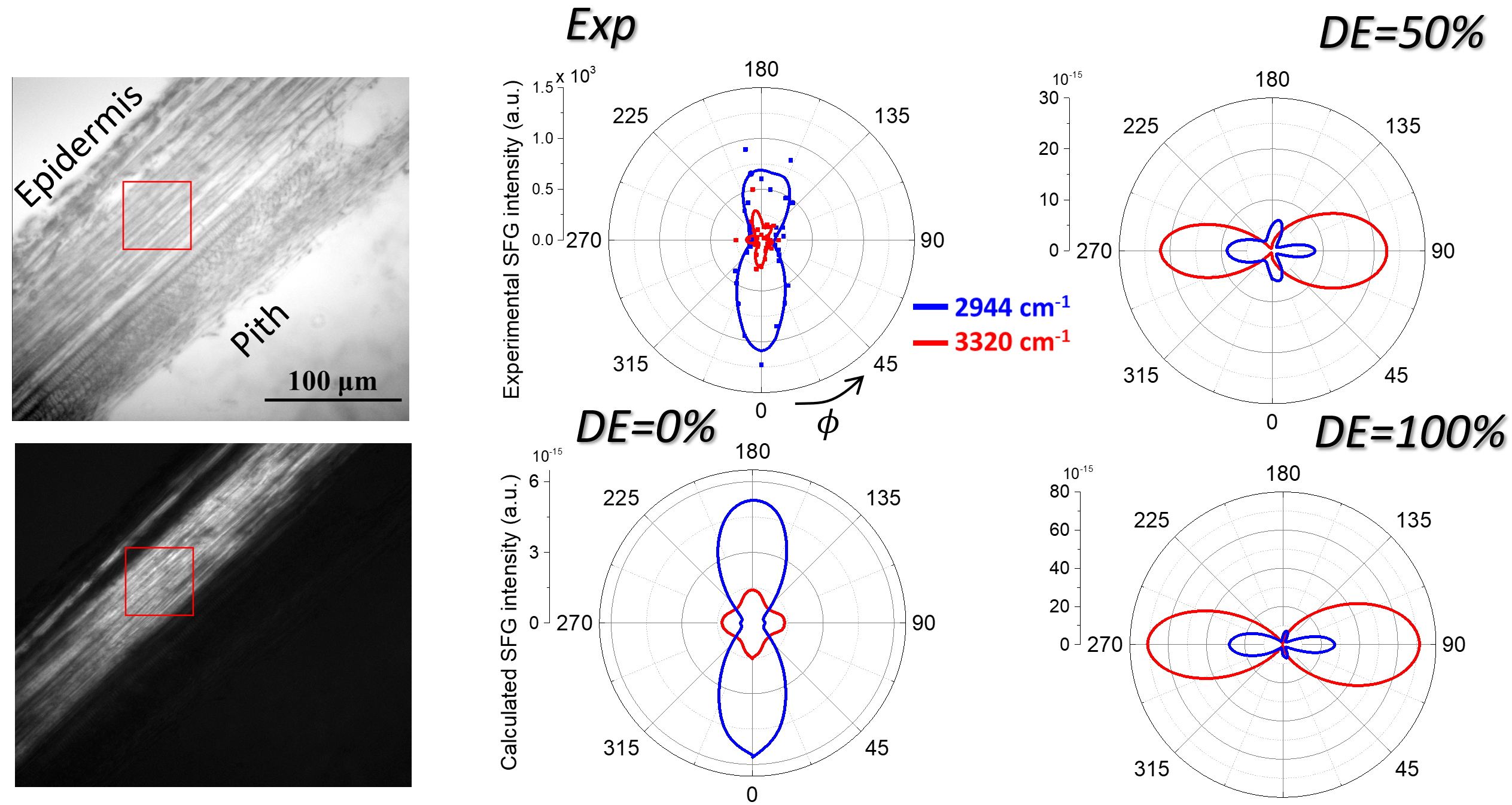
**

Supplementary Figure 4. Optical and cross-polarized light images of a longitudinal section of 8-week-old Arabidopsis stem and the polar plot of the 2944 cm-1 and 3320 cm-1 pps-SFG intensities. Also shown are the polar plots of the same peak intensities calculated for DE = 0%, 50%, and 100% cases at θ = 90°. The dimensions used in the calculation are the same as Fig. 3.


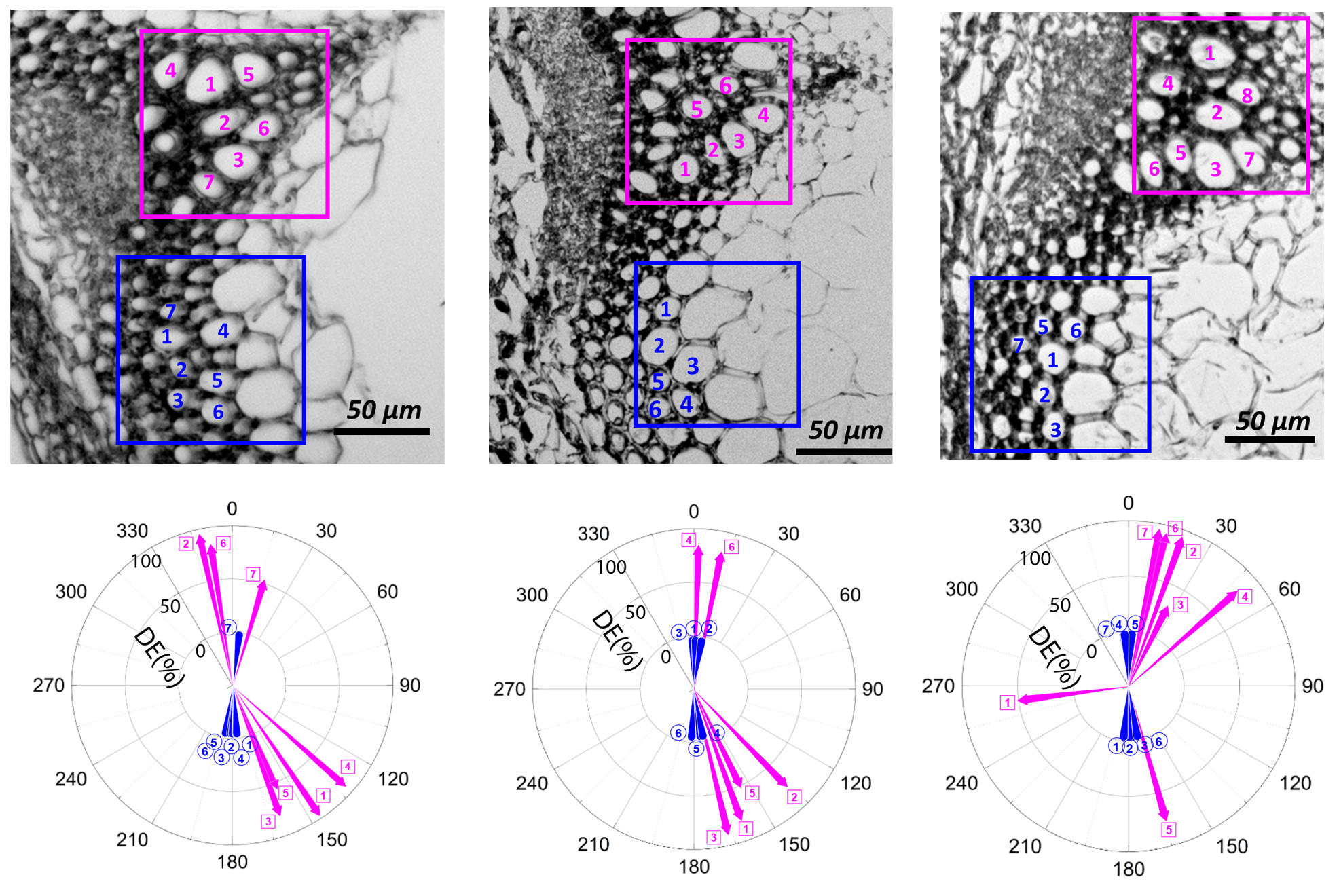


**Supplementary** **Figure 5.** Polar plot of tilt angle (θ) and polarity (DE) of CMF for 20 IFF cells (blue cones) and 20 xylem cells (pink arrows) selected in the SFG hyperspectral images of three individual transverse cross-sections of 8-week-old *Arabidopsis* stems. The direction and length of the arrow mean the tilt (θ) angle and the DE value determined from the MLP method.
